# A multivariate blood metabolite algorithm stably predicts risk and resilience to major depressive disorder in the general population

**DOI:** 10.1016/j.ebiom.2023.104643

**Published:** 2023-06-14

**Authors:** Daniel E. Radford-Smith, Daniel C. Anthony, Fee Benz, James T. Grist, Monty Lyman, Jack J. Miller, Fay Probert

**Affiliations:** a Department of Pharmacology, University of Oxford, Oxford, OX1 3QT, United Kingdom; b Department of Chemistry, University of Oxford, Oxford, OX1 3TA, United Kingdom; c Department of Psychiatry, Warneford Hospital, University of Oxford, Oxford, OX3 7JX, United Kingdom; d Department of Physiology, Anatomy, and Genetics, University of Oxford, Oxford, United Kingdom; e The Oxford Centre for Clinical Magnetic Resonance Research, University of Oxford, Oxford, United Kingdom; f Department of Radiology, Oxford University Hospitals, Oxford, United Kingdom; g Department of Physics, Clarendon Laboratory, Oxford, OX1 3PT, United Kingdom; h The MR Research Centre, Aarhus University, Aarhus, Denmark

**Keywords:** Major depressive disorder, Biobank, Biomarkers, Random forest classification, Pyruvate, Lactate

## Abstract

**Background:**

Socioeconomic pressures, sex, and physical health status strongly influence the development of major depressive disorder (MDD) and mask other contributing factors in small cohorts. Resilient individuals overcome adversity without the onset of psychological symptoms, but resilience, as for susceptibility, has a complex and multifaceted molecular basis. The scale and depth of the UK Biobank affords an opportunity to identify resilience biomarkers in rigorously matched, at-risk individuals. Here, we evaluated whether blood metabolites could prospectively classify and indicate a biological basis for susceptibility or resilience to MDD.

**Methods:**

Using the UK Biobank, we employed random forests, a supervised, interpretable machine learning statistical method to determine the relative importance of sociodemographic, psychosocial, anthropometric, and physiological factors that govern the risk of prospective MDD onset (total n = 15,710). We then used propensity scores to rigorously match individuals with a history of MDD (n = 491) against a resilient subset of individuals without an MDD diagnosis (retrospectively or during follow-up; n = 491) using an array of key social, demographic, and disease-associated drivers of depression risk. 381 blood metabolites and clinical chemistry variables and 4 urine metabolites were integrated to generate a multivariate random forest-based algorithm using 10-fold cross-validation to predict prospective MDD risk and resilience.

**Outcomes:**

In unmatched individuals, a first case of MDD, with a median time-to-diagnosis of 72 years, can be predicted using random forest classification probabilities with an area under the receiver operator characteristic curve (ROC AUC) of 0.89. Prospective resilience/susceptibility to MDD was then predicted with a ROC AUC of 0.72 ($\tilde{x}$ = 3.2 years follow-up) and 0.68 ($\tilde{x}$ = 7.2 years follow-up). Increased pyruvate was identified as a key biomarker of resilience to MDD and was validated retrospectively in the TwinsUK cohort.

**Interpretation:**

Blood metabolites prospectively associate with substantially reduced MDD risk. Therapeutic targeting of these metabolites may provide a framework for MDD risk stratification and reduction.

**Funding:**

New York Academy of Sciences’ Interstellar Programme Award; Novo Fonden; Lincoln Kingsgate award; 10.13039/501100014748Clarendon Fund; Newton-Abraham studentship (10.13039/501100000769University of Oxford). The funders had no role in the development of the present study.

**Research in context**

**Evidence before this study**

Maladaptive stress responses contribute significantly to the risk of depression onset and recurrence, whereas appropriate, non-pathological stress responses are mitigating and decrease risk. While the biological underpinnings of behavioural maladaptation and resilience to chronic stress have been extensively studied in animal models, research in humans is lacking. We searched the MEDLINE database through PubMed for studies published in any language prior to September 2022 regarding biomarkers for depression resilience. Specifically, we searched for “depression AND resilience AND (metabolites OR metabolomics)”. 54 articles were retrieved, of which 39 were solely concerning experiments in rodents, 10 were review articles of low relevance to resilience biology, and the remaining 5 articles were not specific to depression, resilience, or biomarker research. Furthermore, none of the research was conducted prospectively. Additional searches of more general, large-scale biomarker studies and meta-analyses for depression were found to be cross-sectional rather than prospective and were inadequately adjusted for confounders which precludes any inferences of a resilient phenotype.

**Added value of this study**

This the first prospective, large-scale investigation of biomarkers for depression resilience and susceptibility. Using supervised multivariate analysis, we first showed that a large proportion of depression risk in the UK Biobank is explained by lifestyle and physiological (physical disease burden) stressors in combination with psychosocial traits. Using this information to rigorously account for a wide range of socioeconomic, demographic, and comorbid risk factors for depression, we then identified a matched resilient cohort of individuals who were distinguished predominately by elevated blood pyruvate and lactate levels.

**Implications of all the available evidence**

Individuals who demonstrate resilience to major depressive disorder in the face of significant adversity throughout life appear to have a distinct circulating metabolite profile from susceptible individuals. Practically, the subtle manipulation of circulating energy substrates is an achievable goal that could be used prophylactically to enhance resilience to depression.

## Introduction

Major depressive disorder (MDD) is common, pervasive, and a leading global cause of disability.^1^ MDD is chiefly defined as either a sustained depressed mood or a marked loss of interest in most or all of one’s activities, which may coincide with nonspecific somatic symptoms such as fatigue and changes in appetite or body weight.^1^ It is thought to result from a combination of genetic, biological, and psychosocial factors. The complexity of the disorder educes an unpredictable response to current pharmacological and psychological therapies.^2^ Indeed, our understanding of MDD pathophysiology remains limited by our ability to classify individuals who, despite a wide range of aetiological contributing factors, present with largely overlapping physical and psychological symptoms.^3^

While extensive research has highlighted maladaptive responses to stress that contribute to MDD, it remains unclear what biological factors drive appropriate, non-pathological, “resilient” stress responses in the face of significant adversity.^4^ Natural variations in resilience have been studied extensively in animal models of stress-induced depressive-like behaviour,^5^ but translational research in humans is lacking.^4^

In a mass spectrometry metabolomic study, depression was associated with reduced serum levels of laurylcarnitine,^6^ which may relate to altered lipid and energy metabolism in people with depression. Using NMR-based metabolomics, a pooled meta-analysis of >5000 cases of depression and >10,000 controls found significantly altered circulating lipid levels in people with depression, including reduced HDL and increased VLDL and triglyceride.^7^ These and other observational studies, typically cross-sectional in nature, often fail to adequately adjust for socioeconomic covariates that, while important to aetiology, obfuscate the common fundamental pathophysiological basis of the condition. An accurate determination of resilience is therefore precluded.

Metabolomic profiling of blood captures the physiological state of an individual at the time of sampling, and is thought to have significant predictive capacity across a wide range of both neurological and systemic diseases.^8^ The UK Biobank, with up to 15 years of *prospective* individual health records, provides a unique opportunity to enable the generation of matched cohorts that can dissociate intrinsic risk factors from the environmental and socioeconomic factors that are known to influence individual mental health trajectories. A better understanding of these intrinsic risk factors associated with MDD susceptibility and resilience would enable the development of upstream prophylactic interventions that might prove more effective than current treatments.

Here, using a combination of biomarkers and psychosocial risk factors, we first show that MDD susceptibility in the UK Biobank can be predicted with an area under the receiver operator characteristic curve (ROC AUC) of 0.89. We then compared the blood metabolome and biochemistry of resilient and susceptible individuals with similar extrinsic risk factors, and identified a blood-based biomarker panel predictive of MDD resilience. Plasma pyruvate, a key biomarker of resilience, was independently associated with a resilience phenotype in the TwinsUK cohort. This test could serve as a complementary diagnostic approach to stratifying MDD susceptibility, without placing additional strain on tertiary care centres.

## Methods

### Study population and ethics

The UK Biobank is a general population cohort recruited from 22 assessment centres across England, Scotland, and Wales between 2006 and 2010. It comprises 502,411 individuals of middle and old age (range 37–73 years, mean 56.5 years). Written, informed consent was provided by all participants. The UK Biobank has generic ethical approval from the Northwest Multi-centre Research Ethics Committee (ref 11/NW/03820). The current study is registered under the approved research ID 72185.

**Fig. 1:**
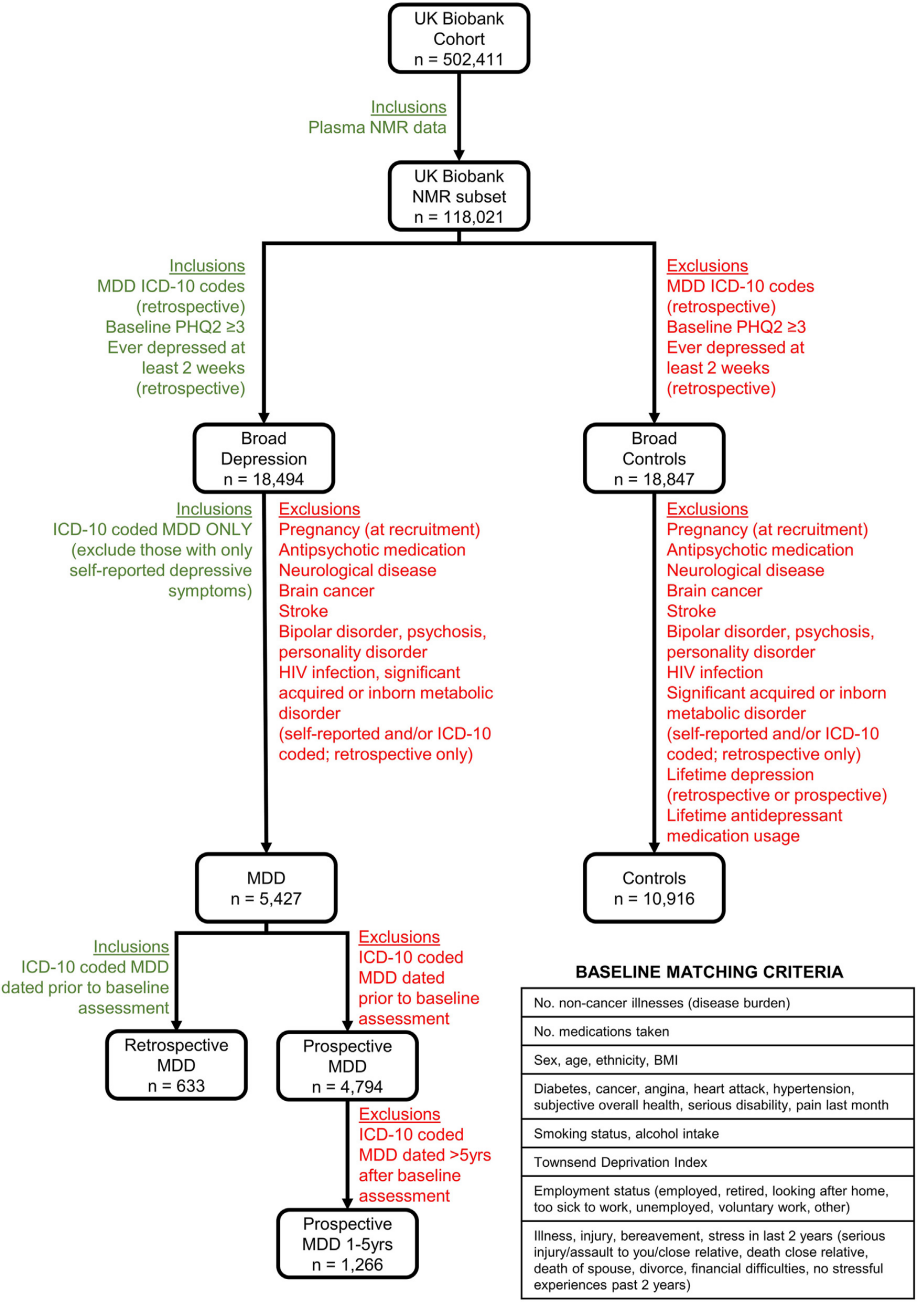
**Flowchart of cohort derivations for control and MDD populations.** Matching criteria are also indicated which were used to derive the matched resilient cohort from the control cohort using propensity scores.

We classified individuals, from the date of blood and urine sampling, as having either a retrospective (n = 2749) or prospective (n = 20,735) diagnosis of International Classification of Diseases (ICD10)-coded MDD according to linked hospital inpatient records. Fig. 1 summarises the exclusion and inclusion criteria for MDD and non-depressed controls, alongside “broad depression” (n = 79,628) and a “broad control cohort” (n = 83,920), and further details are provided in Supplementary Table S1.

### Identifying a population subset resilient to MDD

**Fig. 2:**
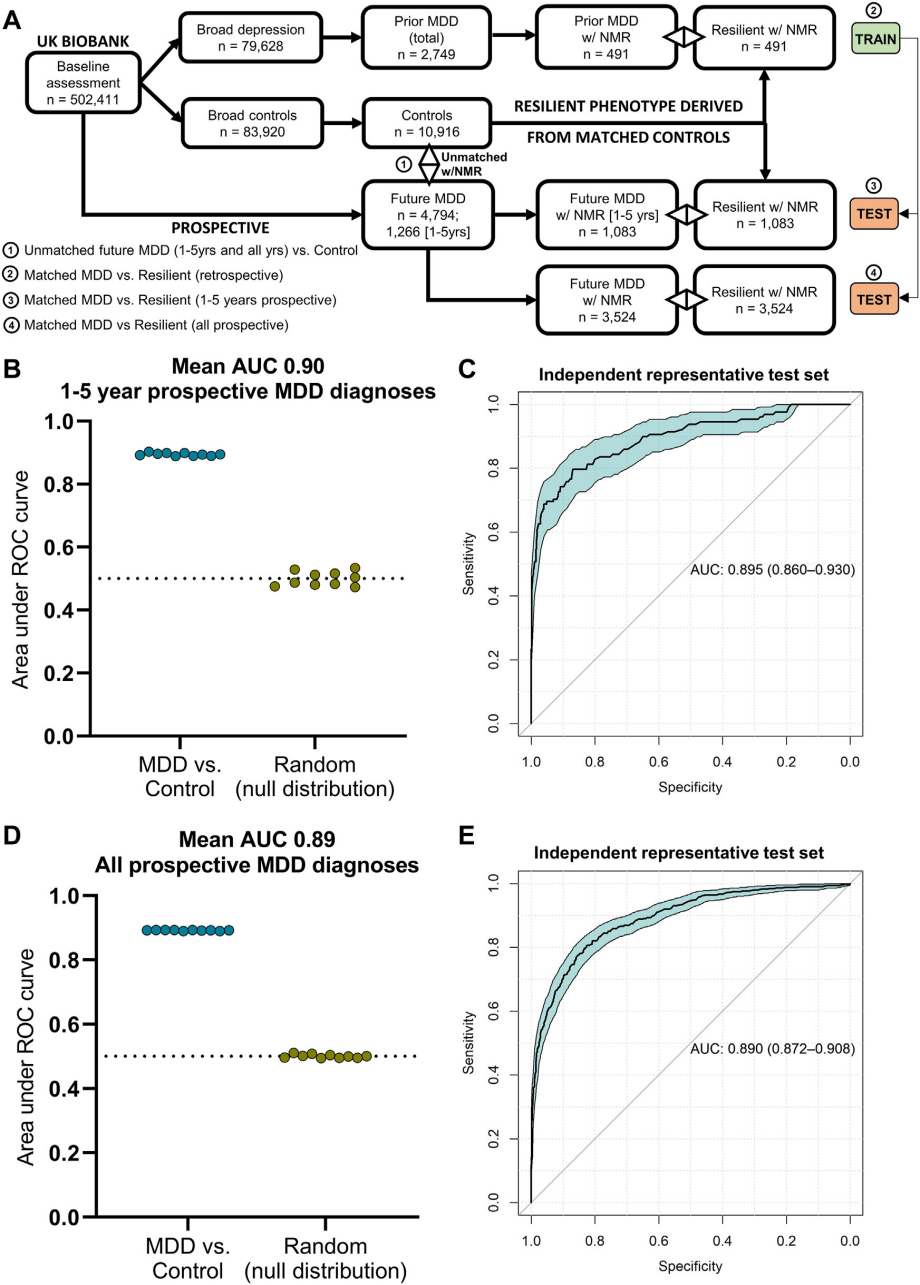
**Machine learning illuminates the anatomy of prospective depression in the UK biobank.** Summary of UK Biobank cohorts (A). Classification rate is high on both matched class sizes with cross validation (B, D) and an unmatched final test set (C, E).

Only those individuals with metabolomic data from the baseline assessment (n = 118,021), corresponding to ∼23% of each cohort, were extracted. From this subset, individuals with an ICD10-coded diagnosis of MDD were matched against controls across a range of socioeconomic, lifestyle, and physiological characteristics (Fig. 1, Fig. 2A and Supplementary Tables S2–S7). Matching was performed using the “MatchIt” package in R v.4.1.3, which aims to produce covariate balance, that is, ensuring that the distributions of covariates in the two groups would approximately be equal to each other, as they would be in a successfully randomized experiment. Propensity scores with a caliper of 0.2 were applied, and each retrospective or prospective MDD case was matched against a single control (1:1 ratio). These matching parameters ensured that no statistically significant differences occurred between MDD and resilient individuals in any of the 33 covariates (Supplementary Tables S5–S7). Control cohorts matched against either retrospective or prospective MDD are referred to as the resilient cohort. A summary of the cohorts used for model training and testing of resilience and susceptibility to MDD in the UK Biobank is shown in Fig. 2A. Further details on the matched cohorts used in this study can be found in the Supplementary methods–resilient cohort derivation in UK Biobank.

*Derivation of susceptible and resilient individuals in the TwinsUK cohort is detailed in the*Supplementary methods–*TwinsUK cohort derivation.*

### Multivariate statistical analysis

Random forest methods^9^ were used to generate algorithms classifying MDD and matched “resilient” or unmatched “control” individuals generated by the procedure above, and using the “randomForest” package in R v.4.1.3.

Prior to analysis by random forest, missing values were imputed using “rfImpute” from “randomForest” in R, and continuous input data was standardised (mean = 0, standard deviation = 1). >99% of individuals had no missing covariate data (Supplementary Table S14) and <2% of the biomarker data required imputation. Missing values from continuous and discrete numerical data were initially imputed as the column median, allowing a random forest model to be generated for the dataset. Imputed observations were then recalculated as the weighted average of the non-missing values, whereby the weights were the proximities from the corresponding random forest proximity matrix. Imputation of categorical data followed a similar procedure. Variables with >2 categories were split into binary variables using “rfImpute” and assigned 0 (absence) or 1 (presence) based on the predicted probabilities using a random forest model.

The parameter mtry, corresponding to the number of random variables selected at each split point for the probabilistic generation of candidate decision trees in the random forest algorithm, serves to reduce overfitting and ensure a diverse representation of features selected at each split in the forest model. Here, mtry was set to the square-root of the number of predictor variables inputted into the model.^10^ The number of trees was fixed in all models at 500.

In the unmatched prospective MDD vs. control models, both covariate data (Supplementary Tables S3 and S4) and blood and urine biomarker data (NMR metabolomics [Category ID 220], urine assays [Category ID 100083], blood count [Category ID 100081], and blood biochemistry [Category ID 17518]) were used as predictor variables. In the matched random forest analysis, only blood and urine biomarker data were used, as the covariate data were used in the matching algorithm.

**Supplementary Fig S1:**
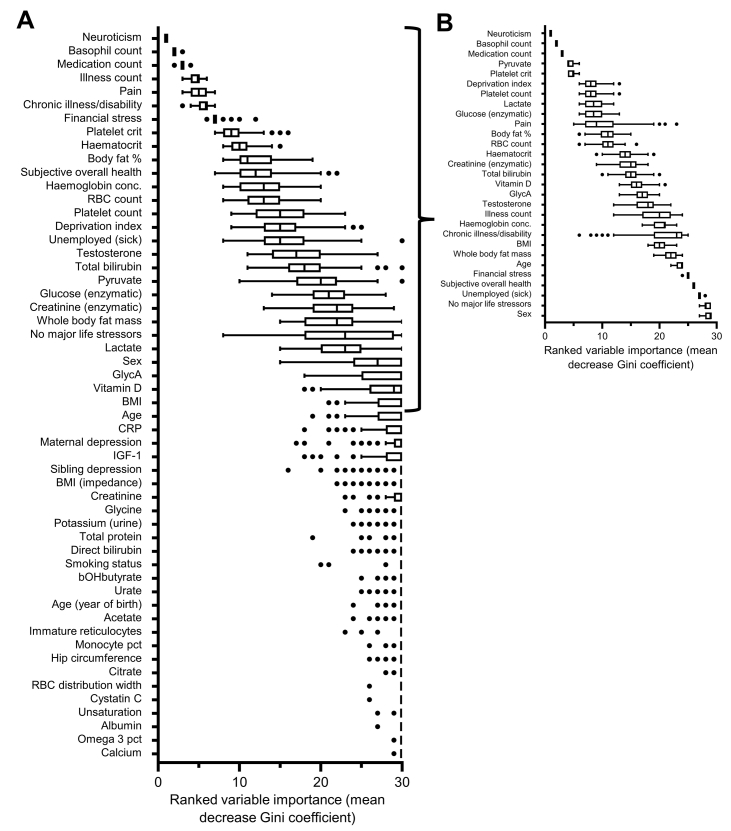


In the unmatched random forest analysis comparing individuals with a prospective 1–5 years MDD diagnosis against controls, data were first split into a training set (90% of individuals) and independent final test set (the remaining 10% of individuals). The training set was subjected to a 10-fold cross-validation procedure to determine whether a model of predictive value could be produced. During this cross-validation, a feature selection step was applied on the training data. Specifically, an initial, “dummy” random forest was produced, from which the top 5% of predictor variables (shown in Table 1 and Supplementary Fig. S1) were shortlisted, and used to generate a second random forest model which was used to test the independent final test set. As the number of control individuals outnumbered those with a prospective MDD diagnosis, a random subset of the control group was extracted prior to each 10-fold cross-validation. This was repeated 10 times, resulting in an ensemble of 100 models from which the mean ROC AUC and most important predictor variables were determined. This process of creating multi-layered random forests is believed to be particularly adept at exploring a larger parameter space quickly in high dimensional data. As a secondary measure to ensure models were not overfitted, a model with randomly permuted classes was fitted and tested in parallel. A mean ROC AUC of ∼0.50 was indicative of a null distribution, to which the predictive models were compared. Finally, the entire training set (90%) was used to predict the independent test set (10%) where class sizes were not matched.

**Table 1:** Summary of the key predictor variables driving prospective MDD risk.

Predictor variable	Variable ID	MDD vs. Control	Representative mean decrease Gini coefficient	
			1–5 yrs	All yrs
Neuroticism score (0–12) *12 questions from the Eysenck personality inventory neuroticism scale*	20127	↑	277.8	883.4
Basophil count	30160	U-shaped association	216.2	747.6
Townsend Deprivation Index (score)	22189	↑	79.1	195.3
No. medications taken (count)	137	↑	77.1	266.2
Glucose	30740	U-shaped association	73.7	194.5
Lactate	23471	↓	69.8	183.6
BMI/WBFM/Body fat %	21001/23100/23099	↑	67.0/62.6/67.7	163.6/159.8/184.1
Pyruvate	23472	↓	65.6	203
Platelet count/crit	30080/30090	↑	65.4/67.3	188.1/206.4
Haematocrit/Haemoglobin concentration/RBC count	30030/30020/30010	↓	64.1/58.7/67.2	173.2/160.6/182.7
Creatinine	30700	U-shaped association	64	174.8
Total bilirubin	30840	↓	63.3	175.1
Glycoprotein acetylation	23480	↑	63.3	169.4
Vitamin D	30890	↓	61.4	173
Testosterone	30850	↓	59.7	167.5
Age	21003	↓	56.1	159.0
Number self-reported non-cancer illnesses (count)	135	↑	54.3	148.4
Cannot work because of illness/disability (%)	6142	↑	48.3	102.1
Self-reported overall (good) health (%)	2178	↓	46.8	98.4
Financial stress (%)	6145	↑	45.4	125.3
Longstanding illness/disability (%)	2188	↑	42.1	165.8
Experienced chronic pain in last month (%)	6159	↑	30.2	145
No/low life stress (%)	6145	↓	13.5	45
Female (%)	31	↑	8.2	37.9

↑ refers to an increase in MDD individuals relative to control individuals; ↓ refers to a decrease in MDD individuals relative to control individuals; U-shaped association refers to an association between both high and low values of the biomarker with an increased risk of MDD relative to control individuals.

For the subsequent random forest analysis classifying all prospective MDD cases against unmatched controls, only the top 50% of feature-selected variables from the 1–5 years prospective analysis were inputted into the model (Supplementary Fig. S1).

**Fig. 3:**
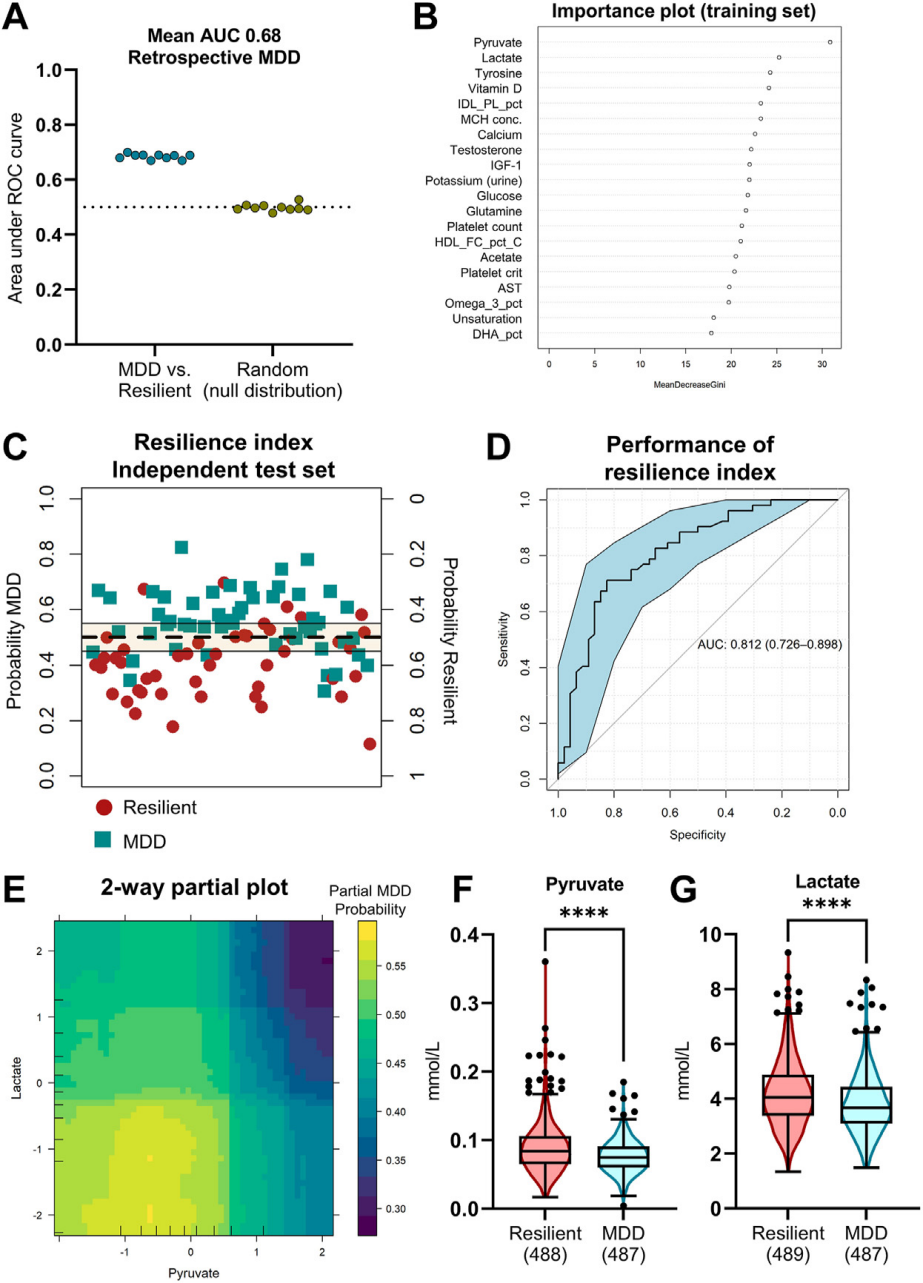
**Elevated plasma pyruvate and lactate drive the resilience phenotype in the UK Biobank.** Matched retrospective MDD and resilient individuals could be discriminated with a mean ROC AUC of 0.68 (A). The training model was driven by pyruvate and lactate (B) and was used to generate a resilience index (C) which achieved a ROC AUC of 0.81 (D). A partial plot from the training algorithm illustrates the probability of being resilient with high circulating lactate and pyruvate levels (E). Individual boxplots (p < 0.0001, *t*-test) are shown in (F) and (G).

Establishing the model distinguishing susceptible (MDD) and resilient individuals in the matched retrospective cohort followed an identical procedure to the prospective 1–5 years analysis, except that only blood (381) and urine (4) biomarkers were used as predictor variables. Selection of predictor variables followed an identical procedure to the unmatched random forest described above, and are shown in Fig. 3B. The random forest algorithm generated from the training set (90%) was then used to predict classification in the matched prospective cohorts.

### Univariate statistical analysis

Univariate data were analysed using Welch’s two-sample *t*-test or, for categorical data, Pearson’s chi-squared test. Bonferroni’s correction was applied throughout, though an unadjusted p > 0.05 was used to conservatively ensure that no differences existed between matched parameters in the matched cohorts. Biomarker significance was interpreted as an adjusted p < 0.05, though Cohen’s *d* measure of effect size was the main outcome of interest. The extreme studentized deviate test was applied to remove any single extreme value present for a biomarker in either group. The log-rank test was used to compare survival curves.

Lastly, all-cause mortality was an additional outcome of interest between individuals who were resilient and susceptible to MDD. Participants who died during the follow-up period (between recruitment and December 2021) were identified from linked NHS death registries which were updated monthly. Associated ICD-10 coded causes of death were categorised and the frequency distribution between resilient and susceptible individuals was analysed using the chi-squared test.

### Role of funding source

This work was supported by the New York Academy of Sciences’ Interstellar Programme Award, Novo Fonden, the Lincoln Kingsgate award, the Clarendon fund, and the Newton-Abraham studentship award (University of Oxford). The funders had no role in the study design, data collection, analyses, or interpretation, and were not involved in the writing or publication process.

## Results

### Machine learning enables prospective MDD classification with 90% certainty

Prospective MDD was accurately classified with a mean cross-validated ROC AUC of 0.90 with negligible error (standard deviation < 0.01; Fig. 2B). The mean out-of-bag (OOB) error for the training set was 0.18. Class-size matching during the 10-fold cross-validation ensured that this accuracy was not due to the lower incidence of MDD. Accordingly, the null distribution (modelled with randomly assigned classes) had a ROC AUC of 0.50 (Fig. 2B). Importantly, a completely independent test set, left out of cross-validation (the remaining 10% of the cohort), was also classified with ROC AUC of 0.90 (95% CI 0.86–0.93, Fig. 2C). Features are shown ranked by importance in Supplementary Fig. S1, and the most important features are shown in Table 1. As expected, neuroticism, physical disease burden, social deprivation, and stressful life events were the top-ranked socioeconomic predictor variables. Increased blood platelet count and glycoprotein acetylation, reduced erythrocytes, testosterone, total bilirubin, pyruvate, lactate, and Vitamin D, and extreme (high or low) levels of basophils, glucose, or creatinine were the top-ranked predictive values in blood. These selected features (Table 1) were subsequently used to distinguish *all* prospective MDD cases (median 7.2 years to diagnosis, 4794 individuals) from the same control cohort.

The cross-validated ROC AUC was 0.89 compared to a null distribution of 0.50 (Fig. 2D), and an independent test set achieved a ROC AUC of 0.89 as well (95% CI 0.87–0.91, Fig. 2E). The mean OOB error for the training set was 0.19. Analysis of the average importance of features during cross-validation revealed some insight into the nature of intrinsic and extrinsic aetiological factors for depression. Baseline neuroticism score, basophil count, and the number of medications being taken remained the top 3 ranked predictors, while self-reported factors including subjective health status and life stressors decreased in relative importance and the plasma biomarkers pyruvate and lactate increased in relative importance (Supplementary Fig. S1). Directionality of these metabolites remained the same, suggesting that the levels of pyruvate and lactate are independent of time to depression and their level at homeostasis confers a degree of resilience or susceptibility to MDD (Table 1).

**Supplementary Fig S2:**
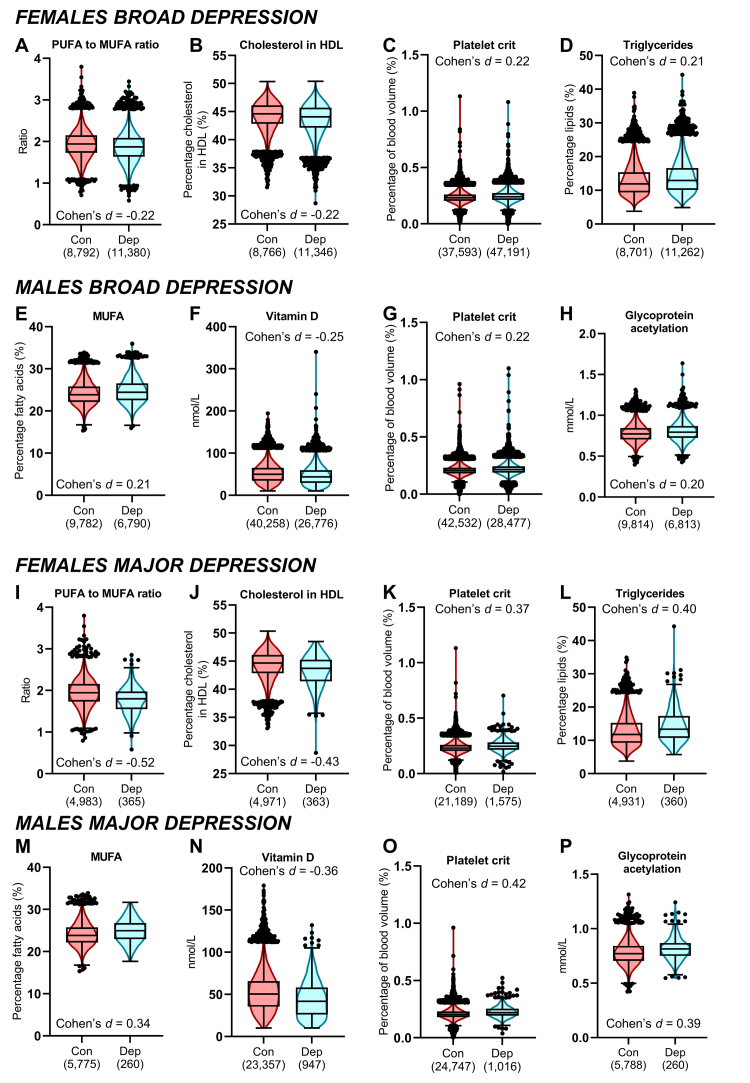


Individuals with a retrospective diagnosis of MDD (n = 633 vs. 10,916 controls), or more broadly, at least two weeks of sustained self-reported depressive symptoms (broad depression, n = 18,494 vs. 18,847 controls) prior to the baseline blood sample confirmed a relationship between depression and glycoprotein acetylation, platelet count, pyruvate, Vitamin D, and RBC levels (Supplementary Fig. S2 and Table S9).

### Resilient individuals have increased pyruvate and lactate levels compared to those with a history of MDD

**Supplementary Fig S3:**
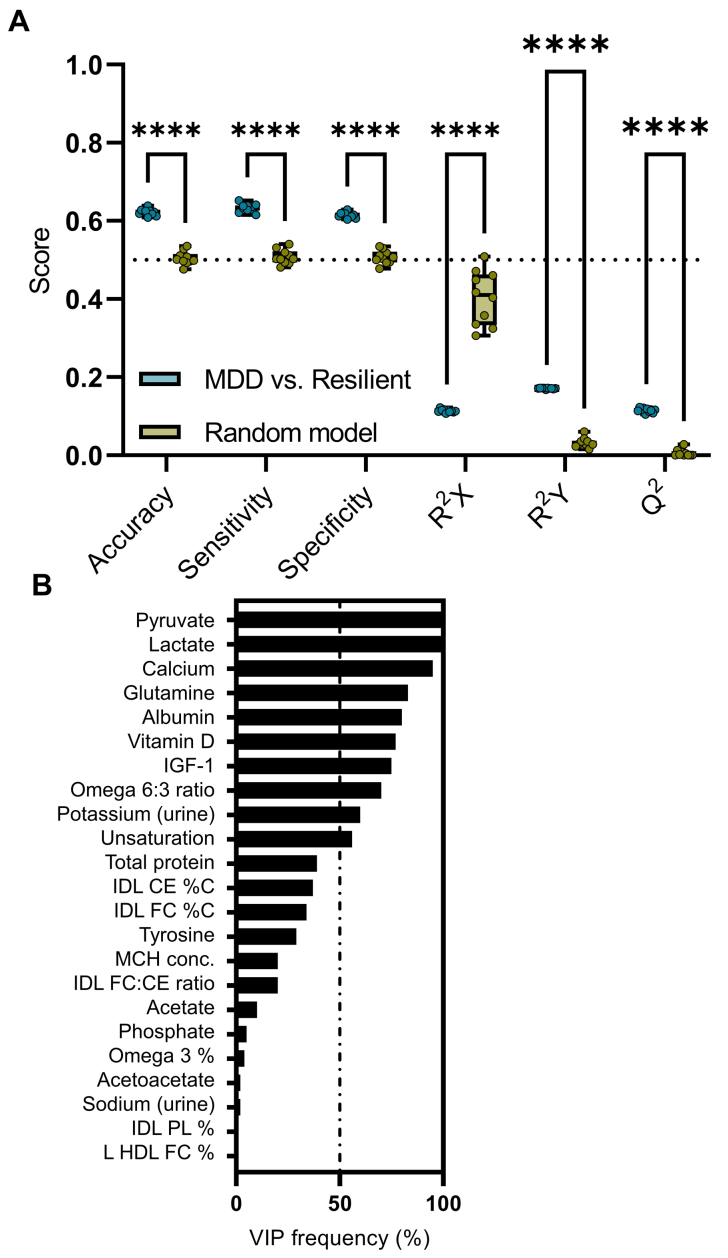


**Supplementary Fig S4:**
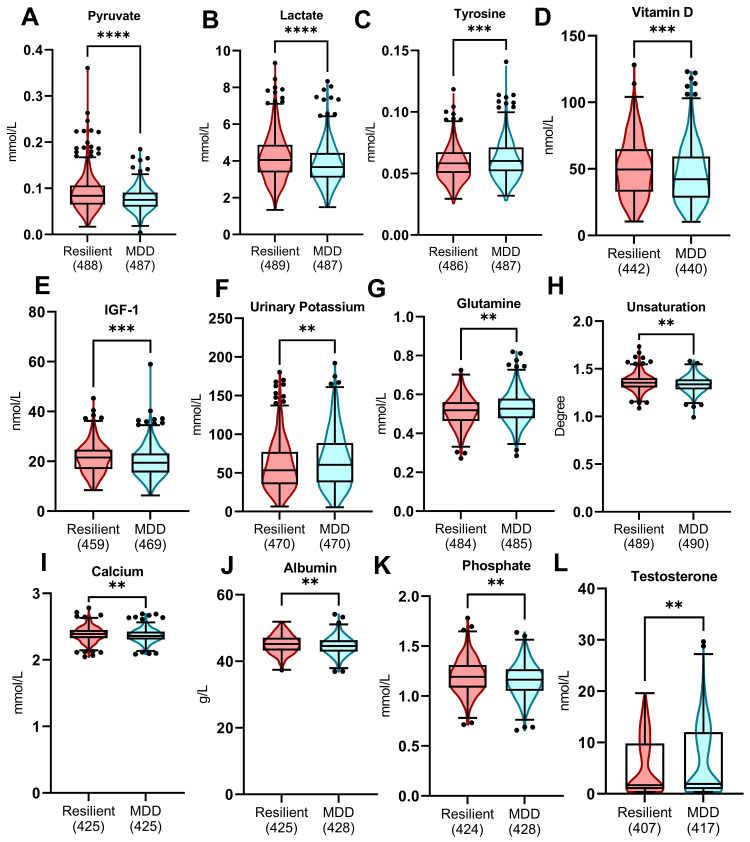


Using 385 biomarkers (381 in blood, 4 in urine), random forest classification with 10-fold cross-validation and 10× repetition (using a training set corresponding to 90% of the matched retrospective cohort) distinguished between retrospective MDD and lifetime resilience with a mean ROC AUC of 0.68 (Fig. 3A). The mean OOB error was 0.37. Inspection of the importance plot revealed a key role of pyruvate in classifying MDD susceptibility and resilience, while lactate was the second identified biomarker (Fig. 3B). A random forest-based algorithm generated from the training set was tested on an independent test set (the remaining 10% of the matched retrospective cohort), classifying MDD with a ROC AUC of 0.81 (95% CI 0.73–0.90, Fig. 3C and D). While a typical random forest machine learning model aggregates multiple decision trees to make predictions in complex datasets, a representative tree^11^ that allows for prediction of MDD and resilient individuals is shown in Supplementary Fig. S11. The accuracy and important features distinguishing susceptibility and resilience were confirmed with an alternate supervised machine learning method (orthogonal partial least squares discriminatory analysis [OPLS-DA] commonly used in metabolomic analyses with multiple colinear covariates), whereby pyruvate and lactate remained the key discriminatory factors (Supplementary Fig. S3). The two-dimensional partial dependence plot of pyruvate and lactate for the random forest model indicates a uniform, linear relationship between concentration and MDD susceptibility across the concentration range (Fig. 3E). Lastly, univariate *t*-testing was performed on all biomarkers in the retrospective cohort. Only pyruvate (Fig. 3F) and lactate (Fig. 3G) remained significant after multiple comparison (Bonferroni’s method, both adjusted p < 0.0001 [*t*-test]). Other biomarkers with a small-to-moderate effect size (Cohen’s *d*) >0.2 are shown in Supplementary Fig. S4.

**Supplementary Fig S5:**
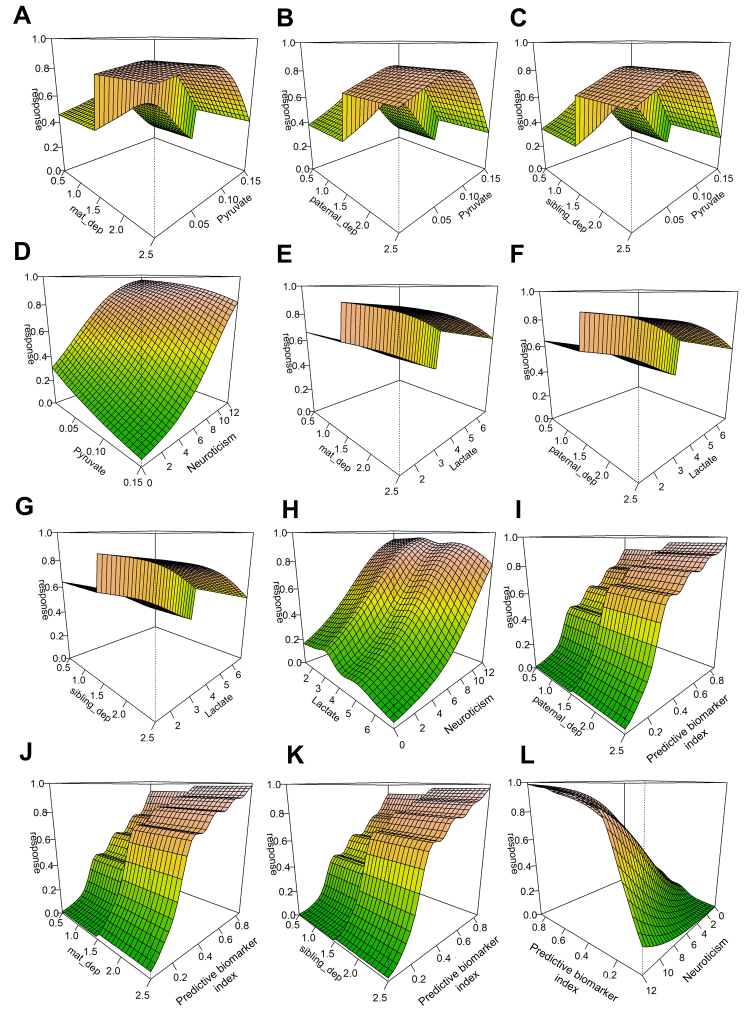


While important covariates such as maternal depression and baseline neuroticism score were not included in the matching algorithm, the MDD risk conferred by pyruvate and lactate concentrations were not found to depend on an individual’s neuroticism score or the presence or absence of familial MDD (Supplementary Fig. S5 and Table S9).

### The metabolomic signature of retrospective MDD susceptibility predicts future MDD onset

**Fig. 4:**
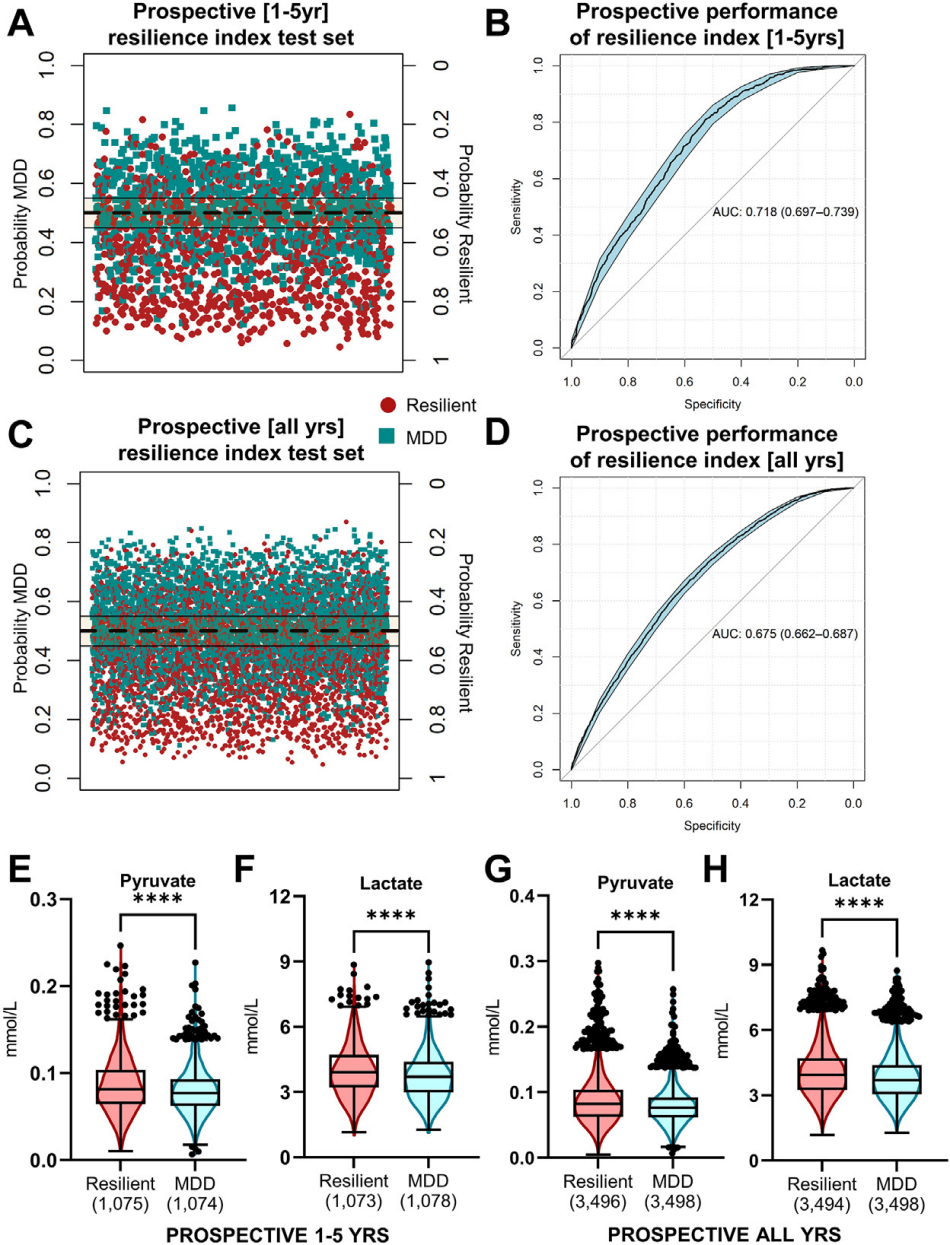
**Prospective MDD susceptibility is predicted from retrospective MDD susceptibility.** Prospectively diagnosed MDD was distinguished from resilient (matched control) individuals, both at 1–5 years follow-up (A, B) and in all prospective cases (C, D). Boxplots of plasma pyruvate and lactate levels (p < 0.0001, *t*-test) are shown for 1–5 years follow-up (E, F) and all prospective cases (G, H).

**Supplementary Fig S6:**
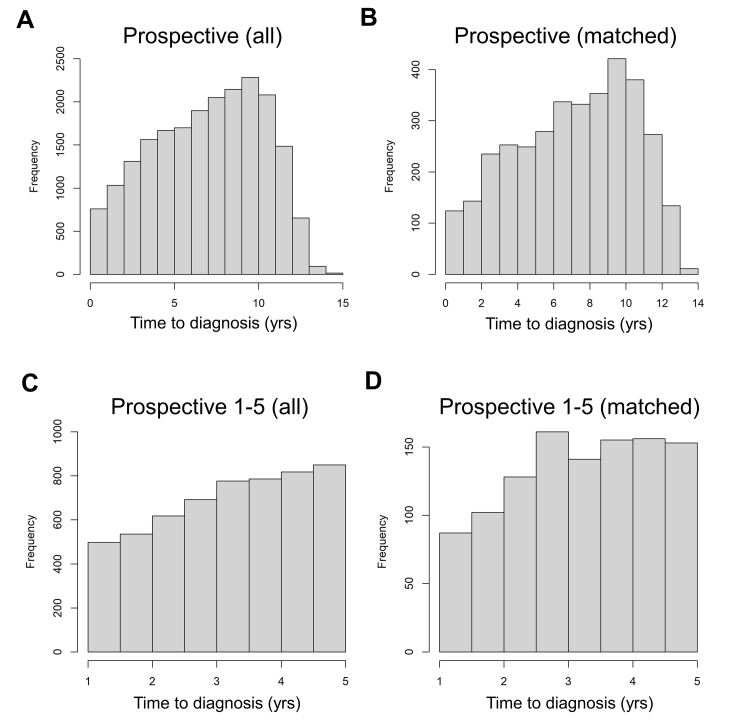


Prospective MDD susceptibility and resilience was predicted with a ROC AUC of 0.72 (95% CI 0.70–0.74, Fig. 4A and B) and 0.68 (95% CI 0.66–0.69, Fig. 4C and D) for a prospective diagnosis between 1 and 5 years and all years of follow-up, respectively. The distribution of follow-up years in each prospective cohort is shown in Supplementary Fig. S6. Plasma pyruvate (Fig. 4E) and lactate (Fig. 4F) were also significantly lower in prospectively susceptible individuals compared to each resilient cohort. In fact, these two metabolites were the only two tested that retained a consistent effect size >0.2 in distinguishing MDD resilience and susceptibility between retrospective and prospective cohorts (Supplementary Table S10).

Pyruvate and lactate levels appeared to be more related to resilience than susceptibility in terms of MDD risk. A basal plasma pyruvate concentration above the ∼75th percentile of the general population (∼0.095 mmol/L) conferred an odds ratio (OR) of 0.38 (95% CI 0.28–0.51) and 0.50 (95% CI 0.45–0.56) for MDD in the retrospective and prospective matched cohorts, respectively. For plasma lactate, a basal concentration above the ∼75th percentile (∼4.3 mmol/L) conferred an OR of 0.50 (95% CI 0.38–0.64) retrospectively and an OR of 0.62 (95% CI 0.56–0.69) prospectively.

### Validation of resilience phenotype in the TwinsUK cohort

**Supplementary Fig S7:**
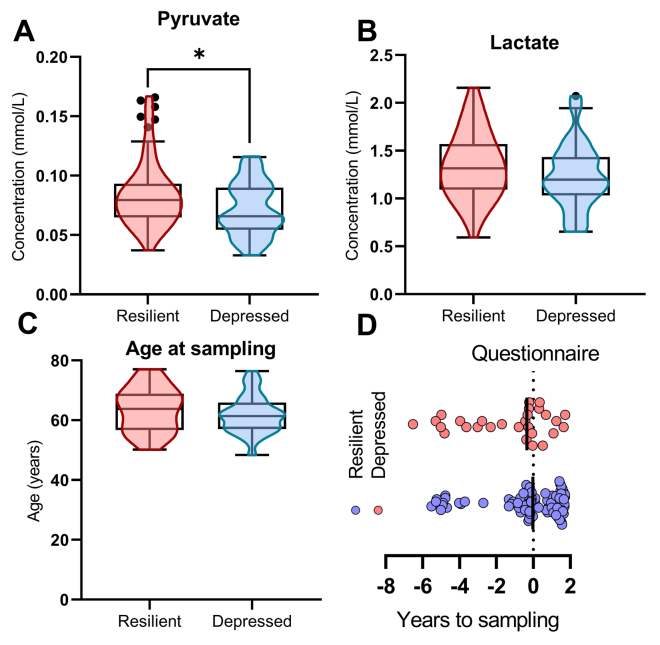


A targeted analysis of pyruvate and lactate levels was performed on a subset of individuals from the TwinsUK biobank. In those with a blood sample most proximal to the questionnaire (3rd visit), serum pyruvate (p = 0.042 [*t*-test], Cohen’s *d* = −0.47 [95% CI −0.91 to −0.04]) was significantly lower in susceptible individuals as compared to resilient individuals (Supplementary Fig. S7A) whilst serum lactate was not significant (p = 0.34 [*t*-test], Cohen’s *d* = −0.32 [95% CI −0.75 to 0.12]; Supplementary Fig. S7B). Blood sampling performed, on average, 6–8 years prior to the questionnaire (visits 1 and 2), did not show a significant difference in pyruvate or lactate level (Supplementary Table S11). Comparisons were adjusted for multiple testing using Bonferroni’s method.

### Individuals with susceptible to MDD are more likely to die prematurely compared to resilient individuals

**Fig. 5:**
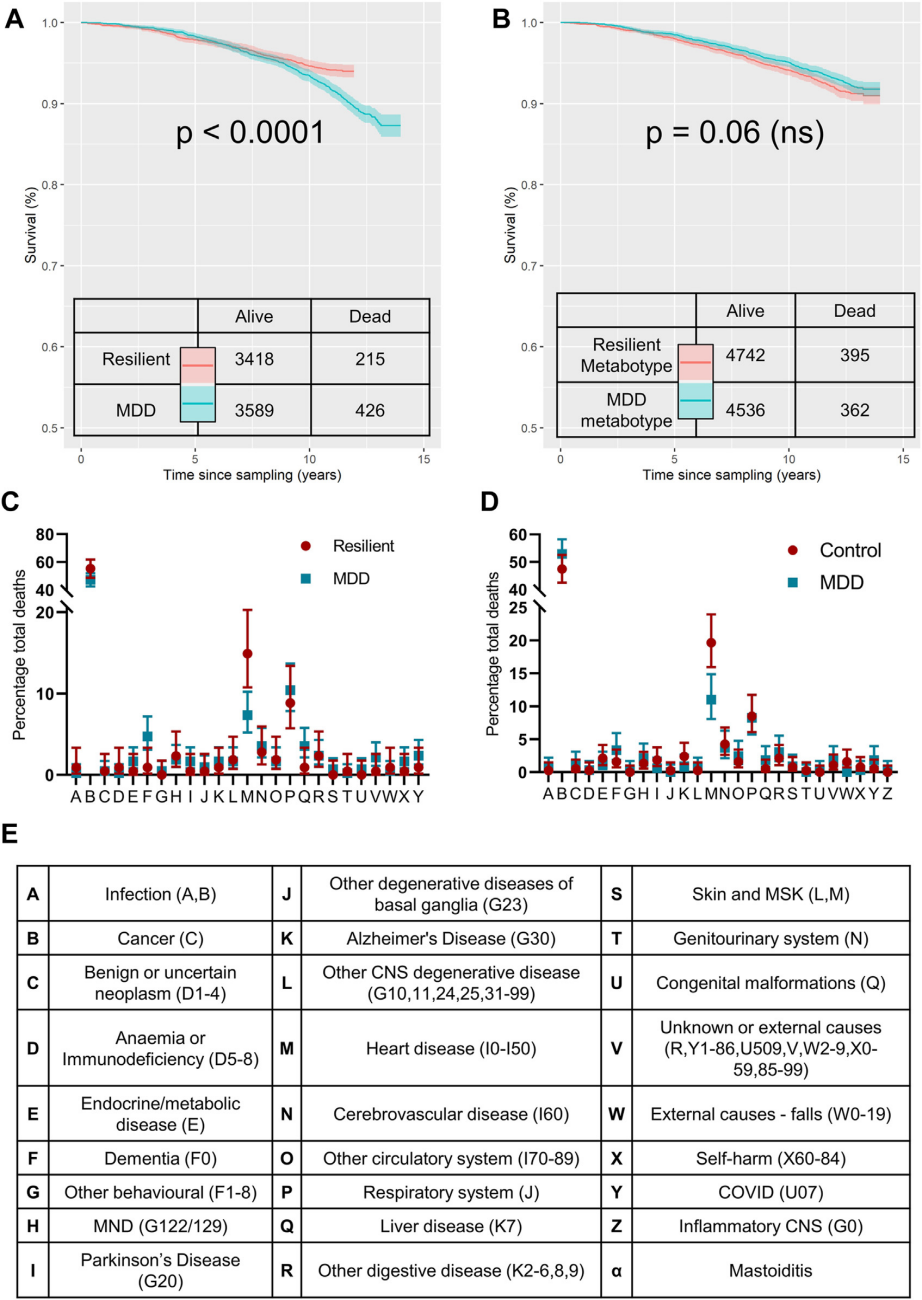
**A metabolomic signature of stress vulnerability predicts premature death.** Kaplan–Meier survival curves shown for resilient and MDD individuals, retrospective or prospective (A), and the metabotype of the general population predicted from the blood biomarker signature of resilience/susceptibility with at least 65% confidence (B). Proportional causes of death are also shown (C, D) with corresponding ICD10 code (E). p-values in (A) and (B) were generated using the log-rank test.

Depression is commonly associated with increased mortality,^12^ though it is unclear whether this association can be attributed solely to the bidirectional relationship between depression and physical disease burden, or socioeconomic status. To this end, we investigated the primary cause of death in those with a lifetime MDD diagnosis and those with a resilient phenotype (the matched cohorts without MDD). Resilient individuals were almost half as likely to have died during the follow-up period (OR 0.53 [95% CI 0.45–0.63]) compared to those with susceptibility to MDD (Fig. 5A). While susceptible individuals were more likely to have died from conditions across the spectrum of disease, a significant difference was identified in the *proportion* of individuals within each group that succumbed to each disease (p = 0.034 [Chi-squared test]). This appeared to be driven by cardiovascular disease, which was relatively lower in those susceptible to MDD, and dementia, which was relatively higher in those with MDD (Fig. 5C and E).

Lastly, we applied the random forest training model used to predict retrospective MDD in Fig. 3B, to determine the health trajectory of those with a metabolomic signature of resilience or susceptibility in the *general population* (n = 117,037). Only those with a prediction confidence of >65% (either towards a resilient or susceptible phenotype) were included in the final analysis, so that individuals with an overtly intermediate metabolite were excluded. 8.6% of individuals within the general population were predicted with this level of confidence and included in the survival curve (Fig. 5B). This was expected given that the blood-based biomarker algorithm was trained in individuals with strong phenotypes (retrospective clinical MDD diagnosis vs. matched resilient controls), not the general population. The odds ratio for death during follow-up was not significantly different between metabotypes in the general population (OR 0.96 [95% CI 0.83–1.11], Fig. 5B). Proportionally, the primary causes of death between those with the different metabotypes were significantly different (p = 0.046 [Chi-squared test]) between those with susceptible and resilient baseline metabotypes (Fig. 5D). Interestingly, these cohort differences followed a similar pattern to the MDD/resilient analysis, whereby proportionally fewer cardiovascular-related deaths occurred in individuals with a MDD-susceptible metabotype.

## Discussion

Here, we utilised the UK Biobank in combination with random forest and propensity score methods to characterise susceptibility and resilience to major depression. We have demonstrated for the first time an association between increased blood pyruvate and lactate, and resilience to MDD in humans. As the key components of a blood-based biomarker algorithm, the circulating concentration of these energy substrates predicted both retrospective and prospective MDD risk and resilience. The association between increased plasma pyruvate and a resilient phenotype was also demonstrated independently in the TwinsUK cohort. Lastly, we were able to demonstrate that, independent of extrinsic factors, MDD susceptibility is associated with premature death across the disease spectrum. Overall, we reveal novel, potentially modifiable biomarkers of altered systemic energy metabolism underpinning MDD risk and resilience.

Until recently, risk factors for MDD have been inextricable from common, confounding features related to allostatic load. For example, a recent, large meta-analyses investigating the blood metabolome in MDD identified fatty acid^13^ and lipoprotein^7^ metabolism as the affected pathways in MDD. While we replicate these findings in our broad and unmatched MDD cohorts, we also show that these metabolic features are unlikely to be independent of covariate physical disease burden and/or socioeconomic status. Furthermore, the association between MDD and inflammation disappeared entirely in our matched cohort (GlycA, CRP Cohen’s *d* < 0.10). Thus, prospective MDD risk in the heterogeneous unmatched cohort could be explained by a combination of life stress, physical disease burden, self-reported health, and neuroticism. This is in keeping with other epidemiological studies, which have identified neuroticism and acute and chronic stressors as key predictors of MDD onset, recurrence, and severity.^14^ However, as the elapsed time between baseline assessment and prospective MDD diagnosis increased, the importance of biological traits became more apparent. Notably, plasma pyruvate concentration increased from a median importance rank of 20 in the 1–5-years prospective random forest, to a median importance rank of 4 when all prospective individuals were assessed, suggesting its homeostatic level may be intrinsically altered in those who are naïve, but highly susceptible to MDD.

In the matched cohort, a metabolomic signature marked chiefly by reduced plasma pyruvate and lactate levels was indicative of prior MDD susceptibility as opposed to lifetime resilience to MDD, and, surprisingly, predicted prospective MDD susceptibility independent of socioeconomic factors and physical disease burden. Our observations do not undermine the extensive research that indicates a key role for psychosocial factors in stress resilience and stress-induced depression.^15^ However, the risk conferred by low pyruvate and lactate levels were independent of these factors, and it is very likely that resilient traits, intrinsic, developmental, or otherwise acquired, have a neurobiological underpinning.^15^

Few studies have investigated biomarkers of MDD resilience in humans, and, to our knowledge, none have done so prospectively. Hitherto, large-scale studies of MDD susceptibility have focused on genome-wide association (GWAS).^16^ Using this approach to understand MDD pathophysiology is problematic. To achieve the power required to inform GWAS, a “broad” phenotype is often used, incorporating many different aetiologies of depression such as a propensity for higher chronic disease burden and other psychopathologies.^16^^,^^17^ Indeed, *NEGR1* has also been associated with obesity through GWAS.^18^ Current polygenic risk scores (PRS) for MDD susceptibility contain upwards of 50,000 risk loci.^14^ While these PRS may achieve predictive power, they are not specific to MDD, do not contribute to insight about underlying disease mechanism, do not consider gene-environment or epigenetic modifications, and are less likely to be modifiable then traits identified at the level of proteins or metabolites.^14^^,^^17^

In terms of physiology, high circulating pyruvate and lactate may indicate increased brain bioavailability of these energy substrates. Blood lactate levels are thought to contribute to 10% of brain energy metabolism at basal lactate levels, and to up to 60% of brain energy metabolism at supraphysiological plasma lactate levels.^19^ Equally, at rest level, the brain is considered to be a net exporter of lactate, with locally produced (astrocytic) brain lactate contributing to the circulating lactate pool.^20^ Increased blood lactate and pyruvate therefore may reflect increased brain metabolic output.^21^

Brain hypometabolism may contribute to stress-induced MDD susceptibility,^22^ whereas lactate and pyruvate are key energy metabolites that serve to maintain brain energy homeostasis.^23^ Peripheral administration of lactate has reduced the effects of chronic stress on depressive-like behaviour in mice,^24^ which, through the production of pyruvate and NADH, promotes neuronal survival and upregulates the expression of synaptic plasticity-related genes.^25^ Conversely, cerebral lactate production from labelled acetate infusion is reduced in chronically stressed animals.^26^ Our laboratory has previously shown that in rodents exposed to probiotic treatment early in life, increased circulating and brain lactate coincided with an increase in resilience to passive stress coping behaviour.^27^ This suggests a possible microbial origin for an increase in the circulating lactate pool.^28^ Overall, there is strong preclinical evidence that lactate and pyruvate maintain energy homeostasis and synaptic plasticity in the adult brain.

Clinically, the high rate of MDD recurrence^29^ observed in the general population is indicative of the low efficacy of current antidepressants. Indeed, up to 50% of individuals with MDD show no response to medical treatment.^4^ Identifying more effective treatments for depression remains a key challenge in the field underpinned by an incomplete understanding of MDD pathogenesis.^2^ Here, the discovery of circulating metabolites associated with MDD presents a new insight into how systemic or brain energy metabolism may contribute to resilient traits. Furthermore, given that these metabolites are known to be brain permeable, neuroactive, and modifiable in concentration, they may also represent therapeutic targets in humans.

For example, metformin, which is the drug of choice for the treatment of type 2 diabetes mellitus via its ability to inhibit gluconeogenesis, also increases circulating levels of lactate.^30^ Metformin, also widely used in psychiatric disorders for the treatment of antipsychotic drug-induced weight gain and insulin resistance,^31^ demonstrates antidepressant-like effects in humans.^32^ While the mechanisms behind this effect remain unclear, the lactate-sparing effect of metformin treatment may play a role.

We recognise the limitations of this study. As an epidemiological resource, the UK Biobank does not include detailed information on depressive traits at the time of recruitment or diagnosis. While others have utilised the more detailed online mental health questionnaire to survey mental health and associated risk factors,^33^ the survey was completed 6–10 years after recruitment and therefore not suitable for contemporaneous biomarker analysis. Rather, we utilised linked ICD-10 codes which indicated a clinical diagnosis, but were relatively nonspecific in terms of MDD severity and subtype (Supplementary Table S12). Similarly, the features used to characterise resilience were, though extensive, not exhaustive. A lack of equivalent covariate data in the TwinsUK cohort precluded replication of the resilience phenotype using the same matching process as the UK Biobank. Further prospective studies of resilience are required to validate the metabolomic phenotype identified here, though the prospective cohort described herein is a powerful approach to address concerns regarding the mediatory role of extrinsic factors that commonly affect cross-sectional studies.

In summary, we have identified circulating lactate and pyruvate as the key components of a biomarker panel for resilience to MDD in the UK Biobank. Subsequently, we identified reduced pyruvate as being significantly associated with MDD susceptibility in the TwinsUK cohort. These two biomarkers, in particular, appear stable, identifying resilience or susceptibility both retrospectively and prospectively, easily quantifiable using standard or NMR-based assays, and show potential for stratifying the susceptible and resilient metabotype of individuals prospectively to maximise efforts towards MDD risk reduction. Future studies would benefit from the inclusion of a framework from which to establish a clear resilience/susceptible phenotype prospectively.

## Contributors

DER-S, DCA, and FP designed the study. DER-S, FP, JJM, and FB analysed the data and verified the data reported in the manuscript and supporting information. DER-S, DCA, JTG, and ML interpreted the data. All authors critically revised the manuscript and approve this version to be published.

## Data sharing statement

The raw biomarker, clinical, and covariate data are available to researchers with an approved project application via www.ukbiobank.ac.uk (UK Biobank) and www.twinsuk.ac.uk (TwinsUK). Detailed descriptive statistics, field IDs, the random forest model and corresponding R code are available in the supplementary information.

## Declaration of interests

No conflicts of interest exist.

## Figures and Tables

**Supplementary Fig S8:**
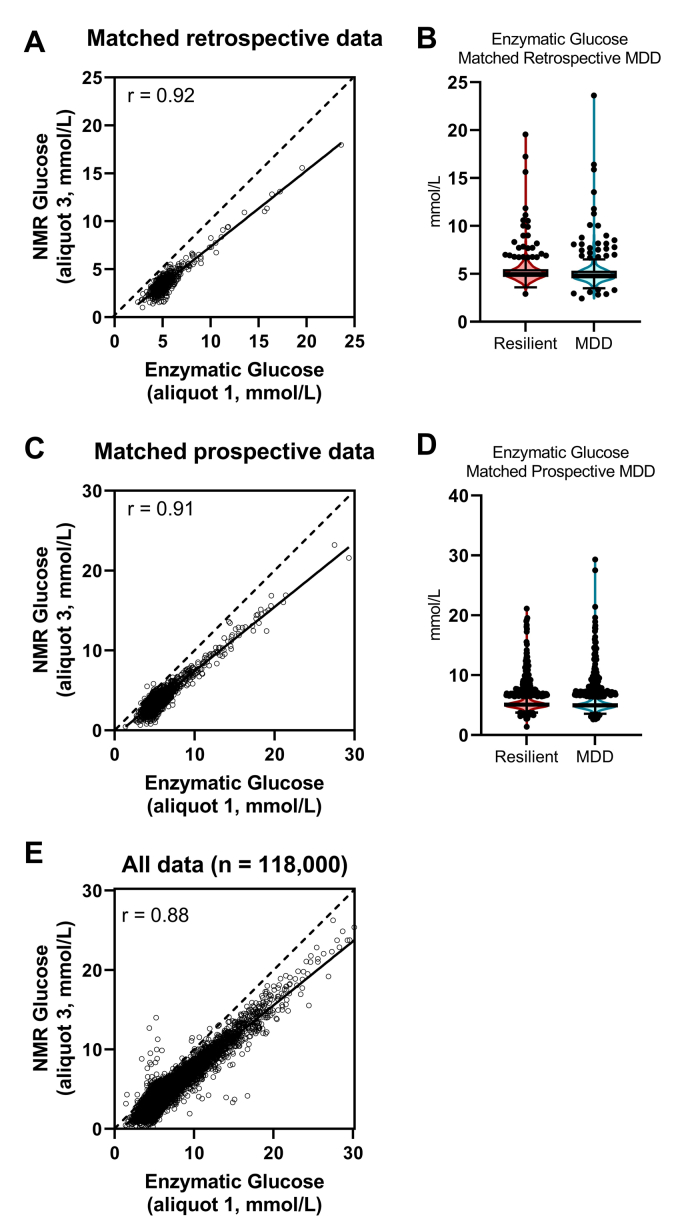


**Supplementary Fig S9:**
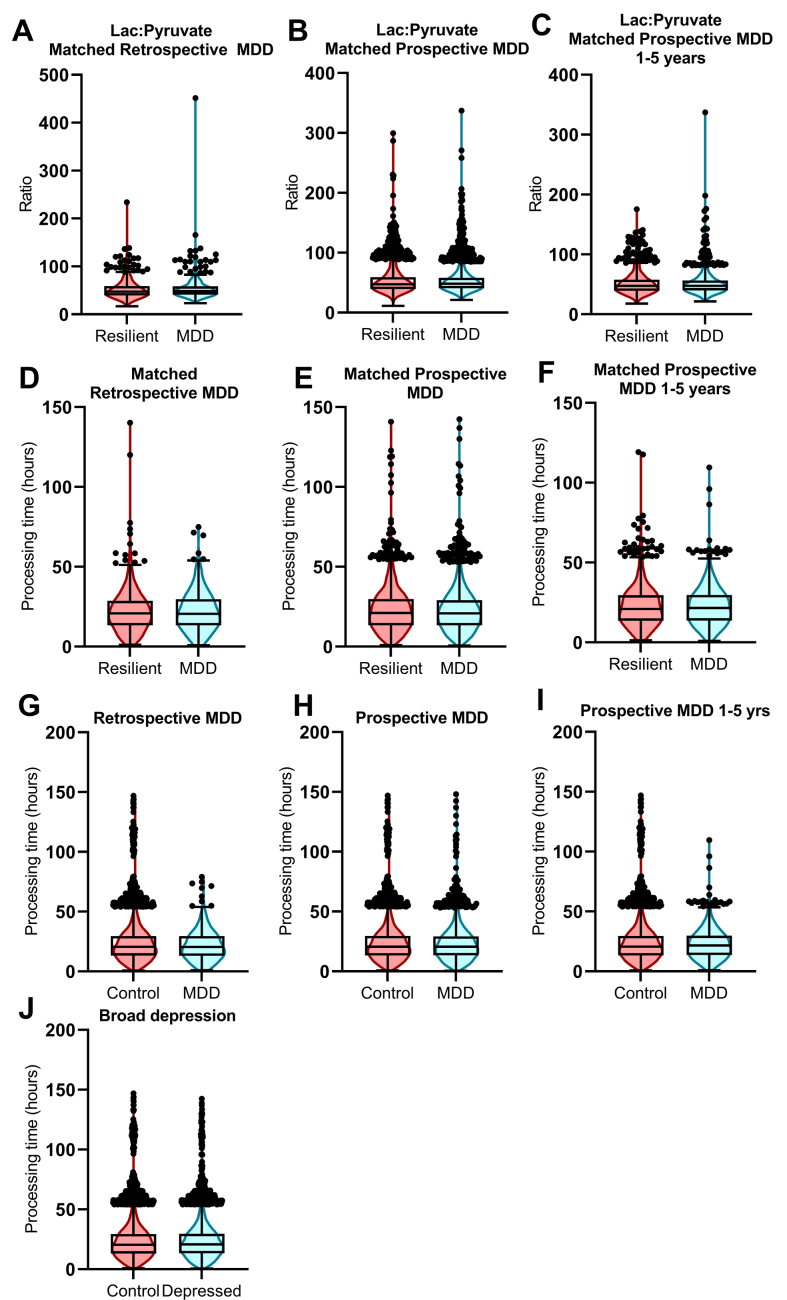


**Supplementary Fig S10:**
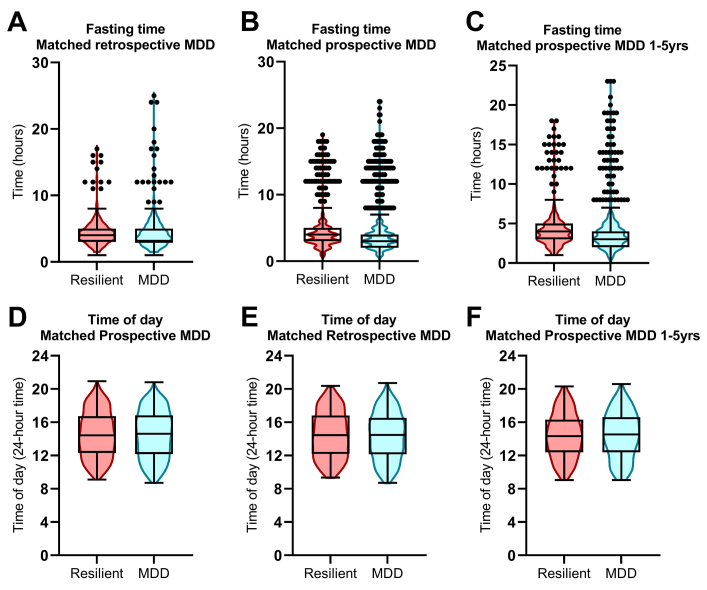


## Appendix A Supplementary data

Supplementary Methods

Supplementary Code

## References

[1] Herrman H., Patel V., Kieling C. (2022). Time for united action on depression: a Lancet–World Psychiatric Association Commission. Lancet.

[2] Malhi G.S., Mann J.J. (2018). Depression. Lancet.

[3] Nandi A., Beard J.R., Galea S. (2009). Epidemiologic heterogeneity of common mood and anxiety disorders over the lifecourse in the general population: a systematic review. BMC Psychiatry.

[4] Krishnan V., Nestler E.J. (2008). The molecular neurobiology of depression. Nature.

[5] Krishnan V., Han M.H., Graham D.L. (2007). Molecular adaptations underlying susceptibility and resistance to social defeat in brain reward regions. Cell.

[6] Zacharias H.U., Hertel J., Johar H. (2021). A metabolome-wide association study in the general population reveals decreased levels of serum laurylcarnitine in people with depression. Mol Psychiatry.

[7] Bot M., Milaneschi Y., Al-Shehri T. (2020). Metabolomics profile in depression: a pooled analysis of 230 metabolic markers in 5283 cases with depression and 10,145 controls. Biol Psychiatry.

[8] Buergel T., Steinfeldt J., Ruyoga G. (2022). Metabolomic profiles predict individual multidisease outcomes. Nat Med.

[9] Richards A. (2015). University of Oxford advanced research computing. https://zenodo.org/record/22558.

[10] Afanador N.L., Smolinska A., Tran T.N., Blanchet L. (2016). Unsupervised random forest: a tutorial with case studies. J Chemom.

[11] Banerjee M., Ding Y., Noone A.M. (2012). Identifying representative trees from ensembles. Stat Med.

[12] Barros V.B., Schmidt F.F.V., Filho A.D.P.C. (2022). Mortality, survival, and causes of death in mental disorders: comprehensive prospective analyses of the UK Biobank cohort. Psychol Med.

[13] Pu J., Liu Y., Zhang H. (2021). An integrated meta-analysis of peripheral blood metabolites and biological functions in major depressive disorder. Mol Psychiatry.

[14] Fang Y., Scott L., Song P., Burmeister M., Sen S. (2020). Genomic prediction of depression risk and resilience under stress. Nat Hum Behav.

[15] Southwick S.M., Vythilingam M., Charney D.S. (2005). The psychobiology of depression and resilience to stress: implications for prevention and treatment. Annu Rev Clin Psychol.

[16] Levey D.F., Stein M.B., Wendt F.R. (2021). Bi-ancestral depression GWAS in the Million Veteran Program and meta-analysis in >1.2 million individuals highlight new therapeutic directions. Nat Neurosci.

[17] Cai N., Revez J.A., Adams M.J. (2020). Minimal phenotyping yields genome-wide association signals of low specificity for major depression. Nat Genet.

[18] Willer C.J., Speliotes E.K., Loos R.J.F. (2009). Six new loci associated with body mass index highlight a neuronal influence on body weight regulation. Nat Genet.

[19] Boumezbeur F., Petersen K.F., Cline G.W. (2010). The contribution of blood lactate to brain energy metabolism in humans measured by dynamic 13C nuclear magnetic resonance spectroscopy. J Neurosci.

[20] van Hall G. (2010). Lactate kinetics in human tissues at rest and during exercise. Acta Physiol.

[21] Schurr A., Miller J.J., Payne R.S., Rigor B.M. (1999). An increase in lactate output by brain tissue serves to meet the energy needs of glutamate-activated neurons. J Neurosci.

[22] Beard E., Lengacher S., Dias S., Magistretti P.J., Finsterwald C. (2022). Astrocytes as key regulators of brain energy metabolism: new therapeutic perspectives. Front Physiol.

[23] Magistretti P.J., Allaman I. (2018). Lactate in the brain: from metabolic end-product to signalling molecule. Nat Rev Neurosci.

[24] Carrard A., Elsayed M., Margineanu M. (2018). Peripheral administration of lactate produces antidepressant-like effects. Mol Psychiatry.

[25] Carrard A., Cassé F., Carron C. (2021). Role of adult hippocampal neurogenesis in the antidepressant actions of lactate. Mol Psychiatry.

[26] Mishra P.K., Kumar A., Behar K.L., Patel A.B. (2018). Subanesthetic ketamine reverses neuronal and astroglial metabolic activity deficits in a social defeat model of depression. J Neurochem.

[27] Radford-Smith D.E., Probert F., Burnet P.W.J., Anthony D.C. (2022). Modifying the maternal microbiota alters the gut-brain metabolome and prevents emotional dysfunction in the adult offspring of obese dams. Proc Natl Acad Sci U S A.

[28] Brooks G.A. (2018). The science and translation of lactate shuttle theory. Cell Metab.

[29] Trivedi M.H., Rush A.J., Wisniewski S.R. (2006). Evaluation of outcomes with citalopram for depression using measurement-based care in STAR∗D: implications for clinical practice. Am J Psychiatry.

[30] Madiraju A.K., Qiu Y., Perry R.J. (2018). Metformin inhibits gluconeogenesis by a redox-dependent mechanism in vivo. Nat Med.

[31] de Silva V.A., Suraweera C., Ratnatunga S.S., Dayabandara M., Wanniarachchi N., Hanwella R. (2016). Metformin in prevention and treatment of antipsychotic induced weight gain: a systematic review and meta-analysis. BMC Psychiatry.

[32] AlHussain F., AlRuthia Y., Al-Mandeel H. (2020). Metformin improves the depression symptoms of women with polycystic ovary syndrome in a lifestyle modification program. Patient Prefer Adherence.

[33] Pitharouli M.C., Hagenaars S.P., Glanville K.P. (2021). Elevated C-reactive protein in patients with depression, independent of genetic, health, and psychosocial factors: results from the UK Biobank. Am J Psychiatry.

