# Supplementary material for: A multivariate blood metabolite algorithm stably predicts risk and resilience to major depressive disorder in the general population
Source: eBioMedicine. 2023 Jun 14;93:104643. doi: 10.1016/j.ebiom.2023.104643 (PMC10275706; doi:10.1016/j.ebiom.2023.104643)
Supplement: Supplementary Table S1 [file mmc1.docx]

**Table S1: Detailed inclusion and exclusion criteria for depression and control cohorts**

| MDD  (retrospective or prospective) | Broad Depression | Broad Control | Control |
| --- | --- | --- | --- |
| INCLUSION MDD ICD10 codes (retrospective = retrospective cohort; prospective = prospective cohort):  "F339","F338","F334", "F332", "F331", "F330", "F341", "F412", "F329", "F328", "F322", "F321", "F320“  AND  Not pregnant at recruitment  AND  No use of antipsychotic medication:  "1141202024", "1141153490", "1141195974", "1140867078", "1140867494", "1141171566", "2038459704", "1140872064", "1140879658", "1140867342", "1140867420", "1140882320", "1140872216", "1140910358", "1141200458", "1141172838", "1140867306", "1140856046", "1140872200", "1140867210", "1140867398", "1140882098", "1140867184", "1140867168", "1140863416", "1140909802", "1140867498", "1140867490", "1140910976", "1140867118", "1140867456", "1140928916", "1140872268", "1140867134", "1140867208", "1140867218", "1140867572", "1140879674", "1140909804", "1140867504", "1140868170", "1140879746", "1141152848", "1141177762", "1140867444", "1140867092", "1141152860", "1140872198", "1140867244", "1140868172", "1140867304", "1140872072", "1140879750", "1140868120", "1140867944", "1140872214", "1141201792", "1140882100", "1141167976”  AND  No self-reported neurological disease:  "1291", "1289", "1082", "1083", "1086", "1524", "1262", "1397", "1683", "1245", "1246", "1491", "1425", "1433", "1258", "1263", "1264", "1266", "1244", "1583", "1659", "1259", "1240", "1434“  AND  No self-reported brain cancers:  "1031", "1032“  AND  No self-reported stroke at baseline  AND  No self-reported bipolar, psychosis, or personality disorder:  "Schizophrenia","Any other type of psychosis or psychotic illness","Mania, hypomania, bipolar or manic-depression","Autism, Asperger's or autistic spectrum disorder","A personality disorder"  AND  EXCLUSION ICD10 codes (only if diagnosed prior to MDD diagnosis): "F309", "F308", "F302", "F301", "F300", "F319", "F318", "F317", "F316", "F315", "F314", "F313", "F312", "F311", "F310", "F29", "F28", "F259", "F258", "F252", "F251", "F250", "F24", "F239", "F238", "F233", "F232", "F231", "F230", "F229", "F228", "F220", "F21", "F209", "F208", "F206", "F205", "F204", "F203", "F202", "F201", "F200","F333", "F323","F448","F009", "F002", "F001", "F000", "F060", "F051", "F03", "F028", "F024", "F023", "F022", "F021", "F020", "F019", "F018", "F013", "F012", "F199", "F198", "F197", "F196", "F195", "F194", "F193", "F192", "F191", "F190", "F620", "F710", "F711", "F718", "F719", "F720", "F721", "F728", "F729", "F730", "F731", "F738", "F739", "F781", "F788", "F791", "F798","G000", "G001", "G002", "G003", "G008", "G009", "G01", "G020", "G021", "G028", "G030", "G031", "G032", "G038", "G039", "G040", "G041", "G042", "G048", "G049", "G050", "G051", "G052", "G058", "G060", "G061", "G062", "G07", "G08", "G09", "G10", "G110", "G111", "G112", "G113", "G114", "G118", "G119", "G120", "G121", "G122", "G128", "G129", "G130", "G131", "G132", "G138", "G14", "G20", "G300", "G301", "G308", "G309", "G310", "G311", "G312", "G318", "G319", "G320", "G328", "G35", "G360", "G361", "G368", "G369", "G370", "G371", "G372", "G373", "G374", "G375", "G378", "G379", "G400", "G401", "G402", "G403", "G404", "G405", "G406", "G407", "G408", "G409", "G800", "G801", "G802", "G803", "G804", "G808", "G809", "G810", "G811", "G819", "G820", "G821", "G822", "G823", "G824", "G825", "G830", "G831", "G832", "G833", "G834", "G835", "G836", "G837", "G838", "G839","B200", "B201", "B202", "B203", "B204", "B205", "B206", "B207", "B208", "B209", "B210", "B211", "B212", "B213", "B217", "B218", "B219", "B220", "B221", "B222", "B227", "B230", "B231", "B232", "B238", "B24","B900", "B901", "B902", "B908", "B909","B180", "B181", "B182", "B188", "B189","D800", "D801", "D802", "D803", "D804", "D805", "D806", "D807", "D808", "D809", "D810", "D811", "D812", "D813", "D814", "D815", "D816", "D817", "D818", "D819", "D820", "D821", "D822", "D823", "D824", "D828", "D829", "D830", "D831", "D832", "D838", "D839", "D840", "D841", "D848", "D849", "D860", "D861", "D862", "D863", "D868", "D869", "D890", "D891", "D892", "D893", "D898", "D899","E000", "E001", "E002", "E009", "E010", "E011", "E012", "E018", "E030", "E031", "E032", "E033", "E034", "E035", "E038", "E039", "E040", "E041", "E042", "E048", "E049", "E050", "E051", "E052", "E053", "E054", "E055", "E058", "E059", "E060", "E061", "E062", "E063", "E064", "E065", "E069", "E070", "E071", "E078", "E079","E40", "E41", "E42", "E43", "E45", "E784", "E780", "E781", "E700", "E701", "E702", "E703", "E708", "E709", "E710", "E711", "E712", "E713", "E720", "E721", "E722", "E723", "E724", "E725", "E728", "E729", "E740", "E741", "E742", "E743", "E744", "E748", "E749", "E750", "E751", "E752", "E753", "E754", "E755", "E756","K754", "K743", "K744", "K745", "K746", "K730", "K731", "K732", "K738", "K739", "K721", "K710", "K711", "K712", "K713", "K714", "K715", "K716", "K717", "K718", "K719" | MDD ICD-10 (left, retrospective only)  AND/OR  Baseline PHQ2 ≥ 3  AND/OR  Baseline ever depressed for at least 2 weeks  AND/OR  Self-reported depression  AND/OR  Probable MDD (single episode or recurrent) | No MDD (retrospective only)  AND  Baseline PHQ2 < 3  AND  Never depressed for a whole week, or ever depressed for less than 2 weeks | Broad Control (left)  AND  Not pregnant at time of recruitment  AND  No use of antipsychotic medication:  "1141202024", "1141153490", "1141195974", "1140867078", "1140867494", "1141171566", "2038459704", "1140872064", "1140879658", "1140867342", "1140867420", "1140882320", "1140872216", "1140910358", "1141200458", "1141172838", "1140867306", "1140856046", "1140872200", "1140867210", "1140867398", "1140882098", "1140867184", "1140867168", "1140863416", "1140909802", "1140867498", "1140867490", "1140910976", "1140867118", "1140867456", "1140928916", "1140872268", "1140867134", "1140867208", "1140867218", "1140867572", "1140879674", "1140909804", "1140867504", "1140868170", "1140879746", "1141152848", "1141177762", "1140867444", "1140867092", "1141152860", "1140872198", "1140867244", "1140868172", "1140867304", "1140872072", "1140879750", "1140868120", "1140867944", "1140872214", "1141201792", "1140882100", "1141167976”  AND  No self-reported neurological disease:  "1291", "1289", "1082", "1083", "1086", "1524", "1262", "1397", "1683", "1245", "1246", "1491", "1425", "1433", "1258", "1263", "1264", "1266", "1244", "1583", "1659", "1259", "1240", "1434“  AND  No self-reported brain cancers:  "1031", "1032“  AND  No self-reported stroke at baseline  AND  No self-reported depression, bipolar, psychosis, or personality disorder:  “Depression”,"Schizophrenia","Any other type of psychosis or psychotic illness","Mania, hypomania, bipolar or manic-depression","Autism, Asperger's or autistic spectrum disorder","A personality disorder"  AND  No lifetime mood disorders:  "F309", "F308", "F302", "F301", "F300", "F319", "F318", "F317", "F316", "F315", "F314", "F313", "F312", "F311", "F310", "F29", "F28", "F259", "F258", "F252", "F251", "F250", "F24", "F239", "F238", "F233", "F232", "F231", "F230", "F229", "F228", "F220", "F21", "F209", "F208", "F206", "F205", "F204", "F203", "F202", "F201", "F200", "F333", "F323","F448","F431", "F412", "F413", "F920", "F928", "F929", "F320", "F321", "F322", "F323", "F328", "F329", "F330", "F331", "F332", "F333", "F334", "F338", "F339", "F340", "F341", "F348", "F349", "F380", "F381", "F388", "F39“  AND  No self-reported mental health issues  "1531", "1286“  AND  No antidepressant medication:  "1140867820", "1140867938", "1140867948", "1140879616", "1140867690", "1141190158", "1141151946", "1140921600", "1140879620", "1141201834", "1140867152", "1140909806", "1140879628", "1140867640", "1141200564", "1141151982", "1140916288", "1141180212", "1140867860", "1140867952", "1140879540", "1140867150", "1140909800", "1140867940", "1140867942", "1140879544", "1140879630", "1140910504", "1140867856", "1140867726", "1140867884", "1140867922", "1140910704", "1140910820", "1140879556", "1141152732", "1140867920", "1140882244", "1140867852", "1140867818", "1141174756", "1140867916", "1140867888", "1140867850", "1140867624", "1140867876", "1141151978", "1140882236", "1140867878", "1201", "1140882312", "1140867758", "1140867712", "1140867914", "1140867944", "1140879634", "1140867756", "1140867934", "1140867960", "1140916282", "1141200570", "1141152736"  AND  No other CNS disease or severe metabolic disease prior to recruitment (same as MDD cohort exclusion ICD10 codes):  "F009", "F002", "F001", "F000", "F060", "F051", "F03", "F028", "F024", "F023", "F022", "F021", "F020", "F019", "F018", "F013", "F012", "F199", "F198", "F197", "F196", "F195", "F194", "F193", "F192", "F191", "F190", "F620", "F710", "F711", "F718", "F719", "F720", "F721", "F728", "F729", "F730", "F731", "F738", "F739", "F781", "F788", "F791", "F798","G000", "G001", "G002", "G003", "G008", "G009", "G01", "G020", "G021", "G028", "G030", "G031", "G032", "G038", "G039", "G040", "G041", "G042", "G048", "G049", "G050", "G051", "G052", "G058", "G060", "G061", "G062", "G07", "G08", "G09", "G10", "G110", "G111", "G112", "G113", "G114", "G118", "G119", "G120", "G121", "G122", "G128", "G129", "G130", "G131", "G132", "G138", "G14", "G20", "G300", "G301", "G308", "G309", "G310", "G311", "G312", "G318", "G319", "G320", "G328", "G35", "G360", "G361", "G368", "G369", "G370", "G371", "G372", "G373", "G374", "G375", "G378", "G379", "G400", "G401", "G402", "G403", "G404", "G405", "G406", "G407", "G408", "G409", "G800", "G801", "G802", "G803", "G804", "G808", "G809", "G810", "G811", "G819", "G820", "G821", "G822", "G823", "G824", "G825", "G830", "G831", "G832", "G833", "G834", "G835", "G836", "G837", "G838", "G839" ,"B200", "B201", "B202", "B203", "B204", "B205", "B206", "B207", "B208", "B209", "B210", "B211", "B212", "B213", "B217", "B218", "B219", "B220", "B221", "B222", "B227", "B230", "B231", "B232", "B238", "B24","B900", "B901", "B902", "B908", "B909","B180", "B181", "B182", "B188", "B189","D800", "D801", "D802", "D803", "D804", "D805", "D806", "D807", "D808", "D809", "D810", "D811", "D812", "D813", "D814", "D815", "D816", "D817", "D818", "D819", "D820", "D821", "D822", "D823", "D824", "D828", "D829", "D830", "D831", "D832", "D838", "D839", "D840", "D841", "D848", "D849", "D860", "D861", "D862", "D863", "D868", "D869", "D890", "D891", "D892", "D893", "D898", "D899","E000", "E001", "E002", "E009", "E010", "E011", "E012", "E018", "E030", "E031", "E032", "E033", "E034", "E035", "E038", "E039", "E040", "E041", "E042", "E048", "E049", "E050", "E051", "E052", "E053", "E054", "E055", "E058", "E059", "E060", "E061", "E062", "E063", "E064", "E065", "E069", "E070", "E071", "E078", "E079","E40", "E41", "E42", "E43", "E45", "E784", "E780", "E781", "E700", "E701", "E702", "E703", "E708", "E709", "E710", "E711", "E712", "E713", "E720", "E721", "E722", "E723", "E724", "E725", "E728", "E729", "E740", "E741", "E742", "E743", "E744", "E748", "E749", "E750", "E751", "E752", "E753", "E754", "E755", "E756","K754", "K743", "K744", "K745", "K746", "K730", "K731", "K732", "K738", "K739", "K721", "K710", "K711", "K712", "K713", "K714", "K715", "K716", "K717", "K718", "K719" |
