# Supplementary figures and images for: A multivariate blood metabolite algorithm stably predicts risk and resilience to major depressive disorder in the general population
Source: eBioMedicine. 2023 Jun 14;93:104643. doi: 10.1016/j.ebiom.2023.104643 (PMC10275706; doi:10.1016/j.ebiom.2023.104643)

**
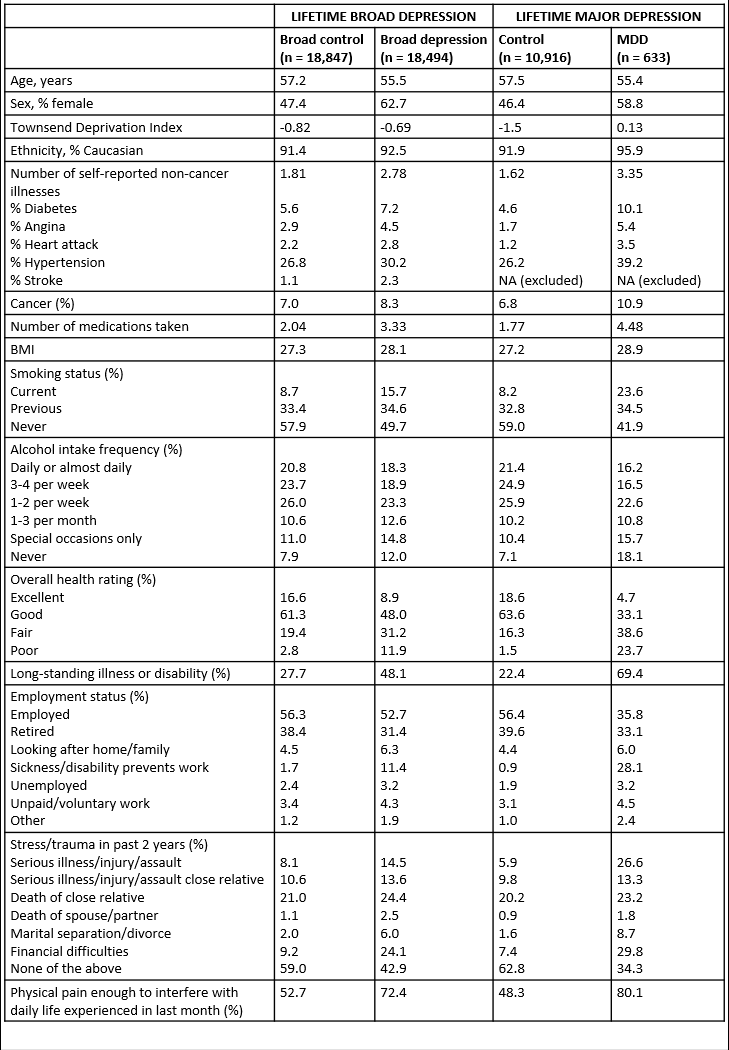
****Table S2: Characteristics of broad and clinical depression compared to (unmatched) controls**

Supplement: Supplementary Table S2 [file mmc2.docx]

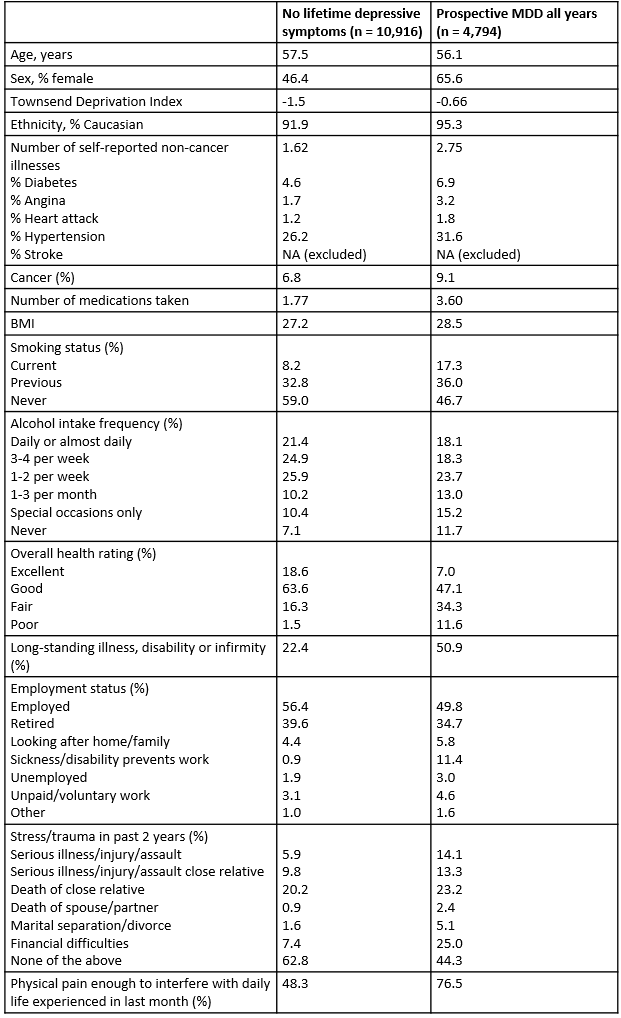
**Table S3: Characteristics of prospective clinical depression compared to (unmatched) controls**

Supplement: Supplementary Table S3 [file mmc3.docx]

**Table S5: Characteristics of matched retrospective cohort**


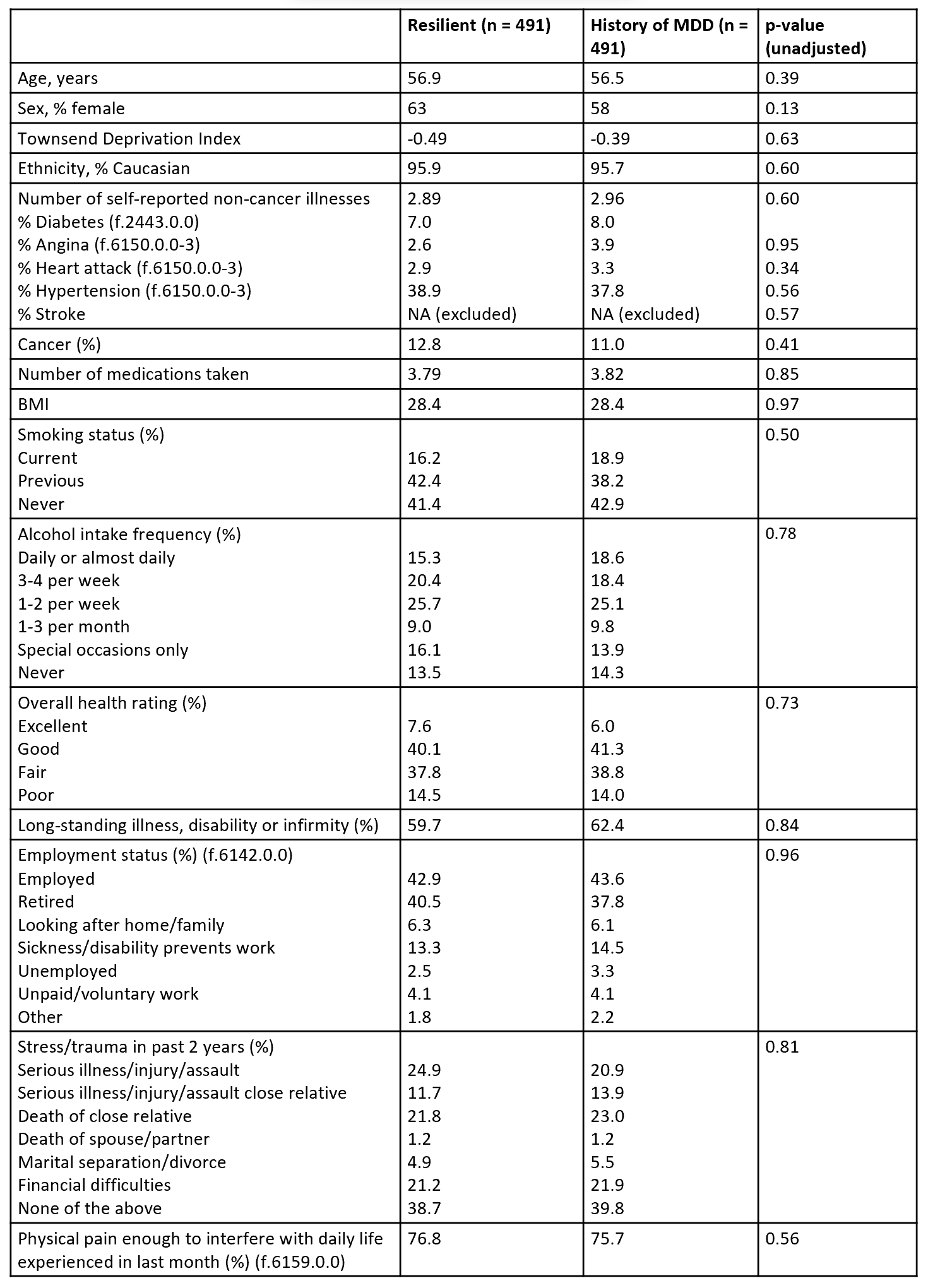

Supplement: Supplementary Table S5 [file mmc5.docx]

**Table S6: Characteristics of matched prospective cohort**


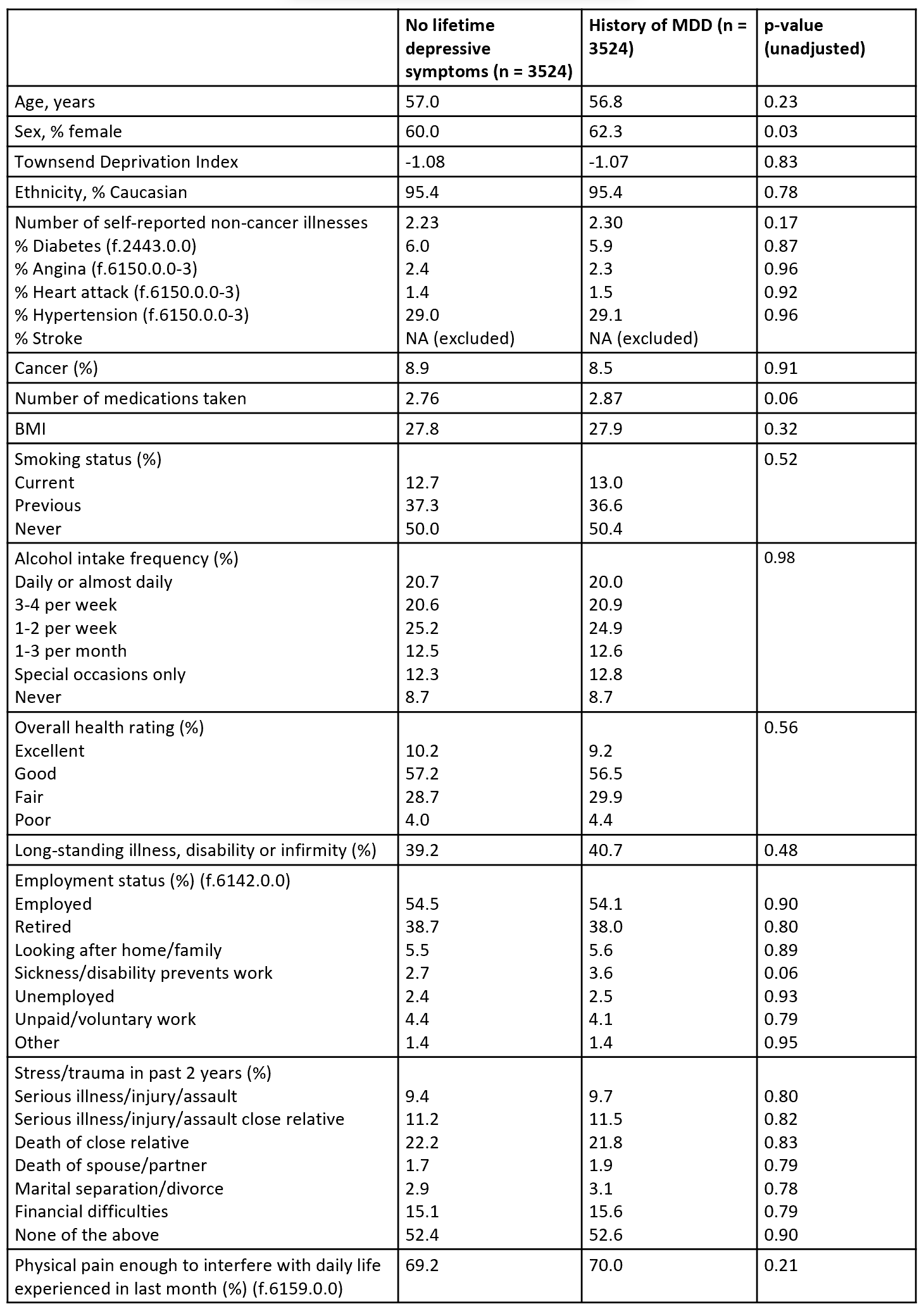

Supplement: Supplementary Table S6 [file mmc6.docx]

**Table S7: Characteristics of matched prospective cohort (1-5 year subset)**


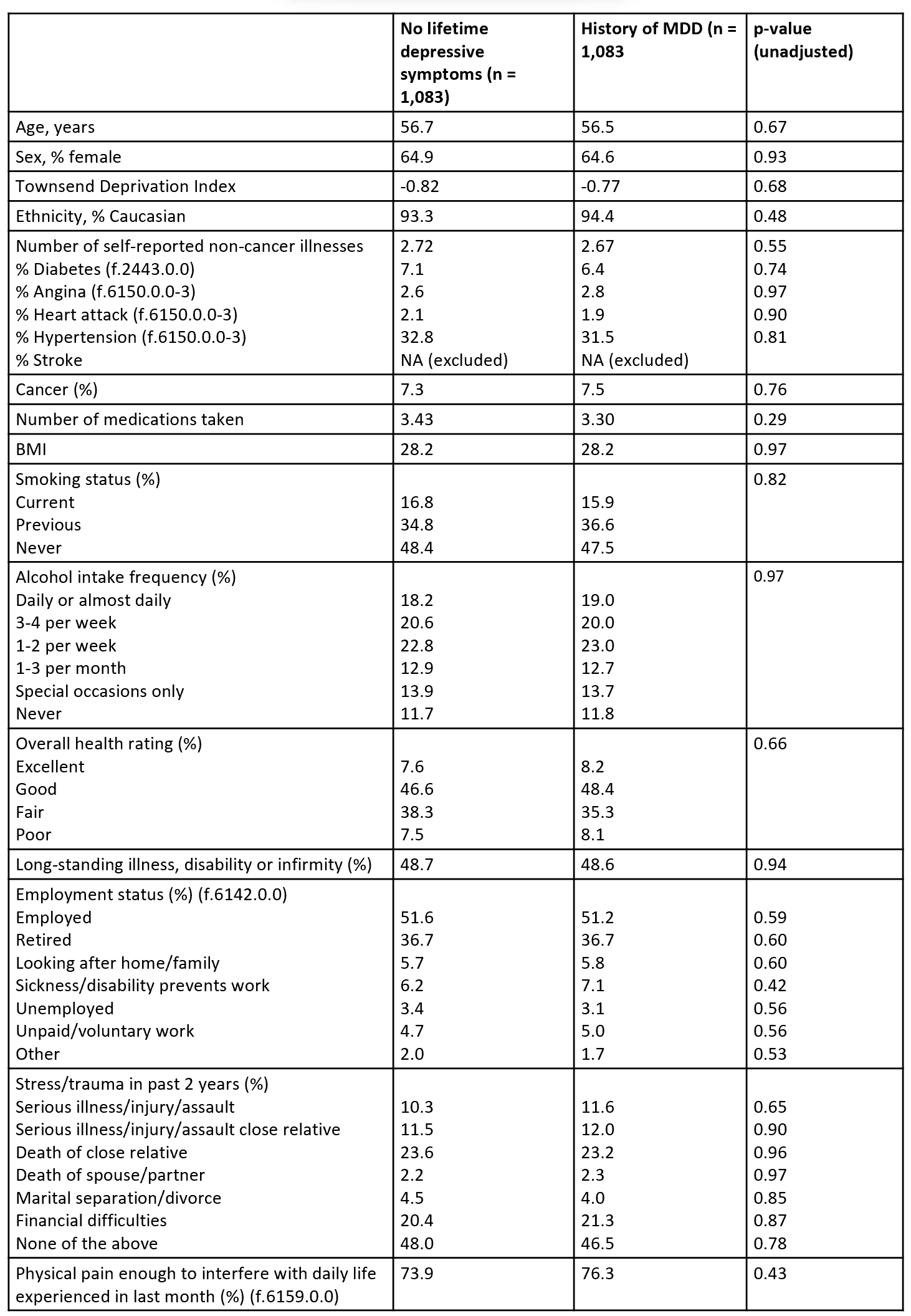

Supplement: Supplementary Table S7 [file mmc7.docx]

**Table S13: Baseline cognitive function in matched cohorts**


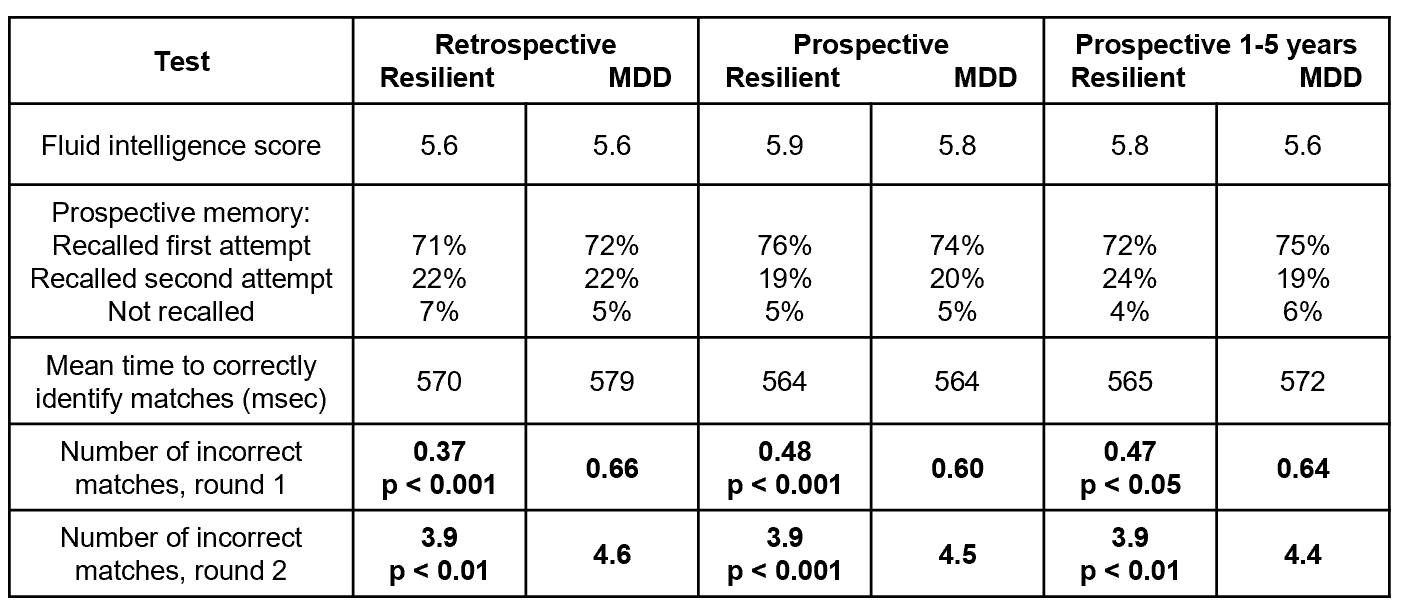

Supplement: Supplementary Table S13 [file mmc13.docx]
