# Supplementary material for: A multivariate blood metabolite algorithm stably predicts risk and resilience to major depressive disorder in the general population
Source: eBioMedicine. 2023 Jun 14;93:104643. doi: 10.1016/j.ebiom.2023.104643 (PMC10275706; doi:10.1016/j.ebiom.2023.104643)
Supplement: Supplementary Table S4 [file mmc4.docx]

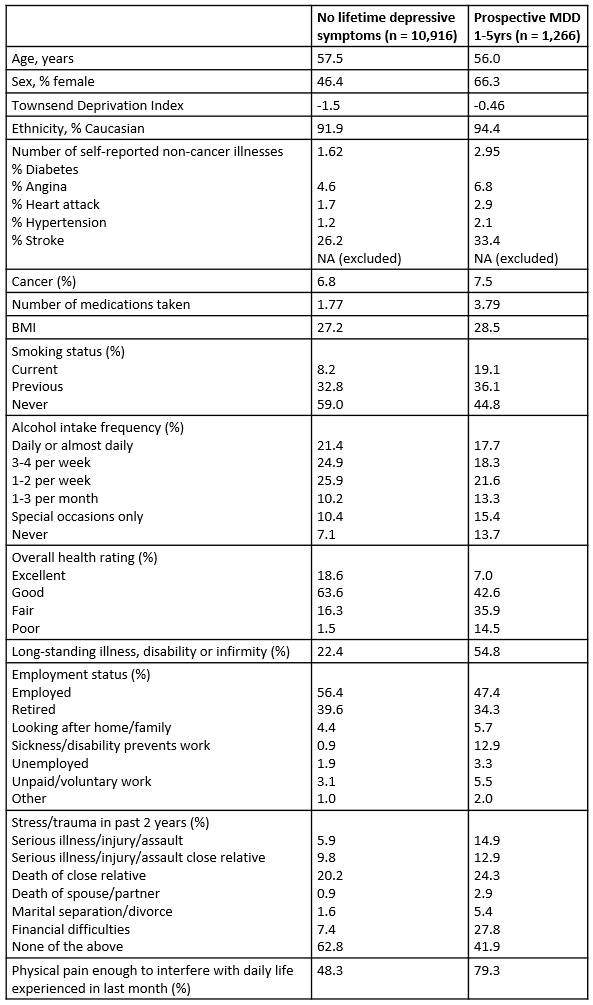
**Table S4: Characteristics of prospective clinical depression (1-5 years only) compared to (unmatched) controls**
