# Supplementary material for: A multivariate blood metabolite algorithm stably predicts risk and resilience to major depressive disorder in the general population
Source: eBioMedicine. 2023 Jun 14;93:104643. doi: 10.1016/j.ebiom.2023.104643 (PMC10275706; doi:10.1016/j.ebiom.2023.104643)
Supplement: Supplementary Table S8 [file mmc8.docx]

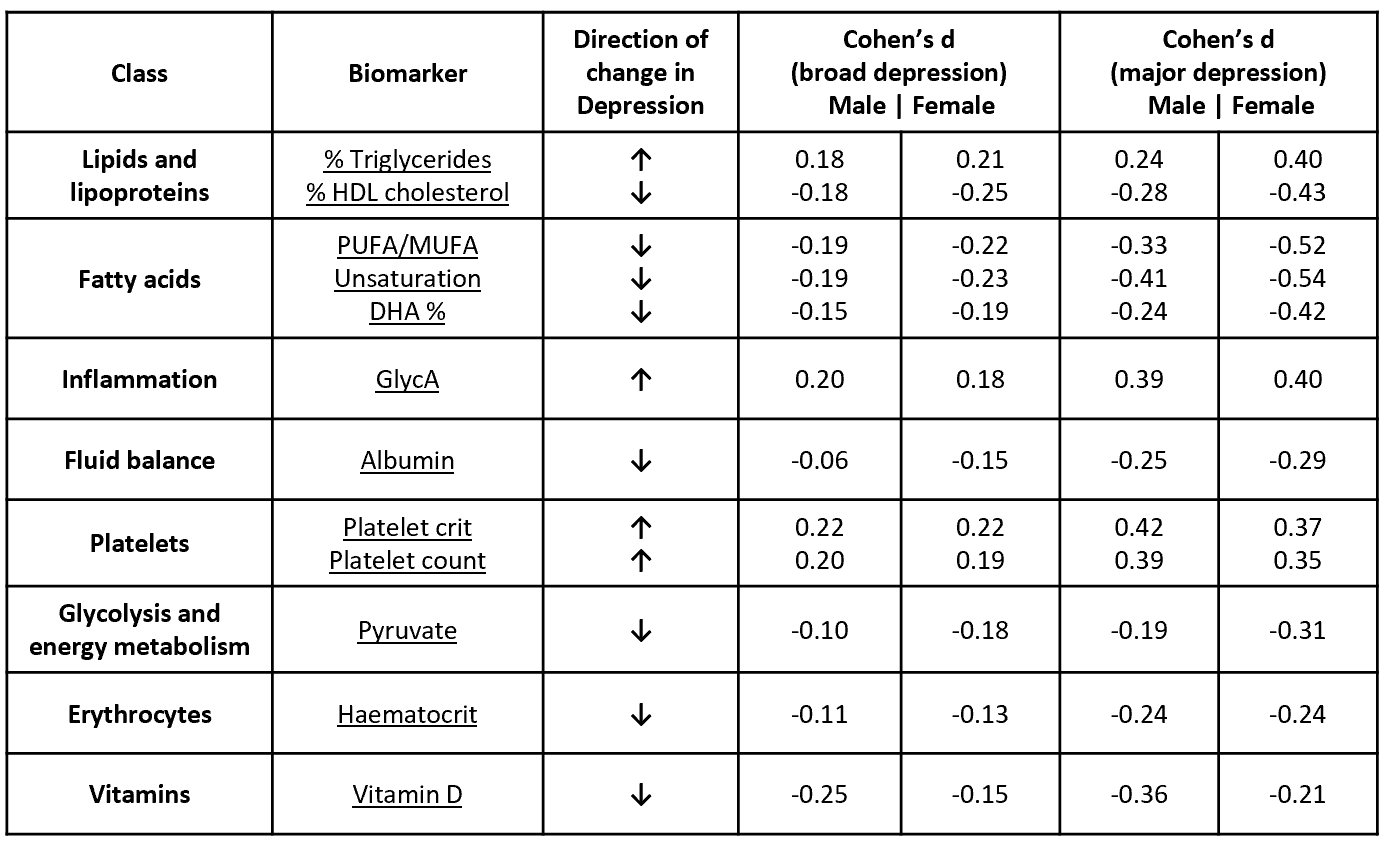
**Table S8: Summary of key biomarkers from retrospective analysis of broad depression and major depression**
