# Supplementary material for: A multivariate blood metabolite algorithm stably predicts risk and resilience to major depressive disorder in the general population
Source: eBioMedicine. 2023 Jun 14;93:104643. doi: 10.1016/j.ebiom.2023.104643 (PMC10275706; doi:10.1016/j.ebiom.2023.104643)
Supplement: Supplementary Table S9 [file mmc9.docx]

**Table S9: Summary of multiple logistic regression interaction terms and statistical significance between pyruvate, lactate, the predictive biomarker index model and selected covariates on the outcome variable (MDD vs. resilience)**

|  | **Maternal depression** | **Sibling depression** | **Paternal depression** | **Neuroticism** |
| --- | --- | --- | --- | --- |
| **Pyruvate** | Estimate: -11.25  Std Error: 16.16  p = 0.49 | Estimate: -11.76  Std Error: 11.06  p = 0.29 | Estimate: -29.12  Std Error: 25.48  p = 0.25 | Estimate: -1.51  Std Error: 1.04  p = 0.15 |
| **Lactate** | Estimate: 0.067  Std Error: 0.25  p = 0.79 | Estimate: -0.016  Std Error: 0.22  p = 0.94 | Estimate: -0.13  Std Error: 0.47  p = 0.78 | Estimate: -0.01  Std Error: 0.024  p = 0.75 |
| **Predictive biomarker index** | Estimate: 196.7  Std Error: 20044.4  p = 0.99 | Estimate: -6.14  Std Error: 32864.3  p = 0.99 | Estimate: 171.7  Std Error: 20238.6  p = 0.99 | Estimate: 1.20  Std Error: 1.18  p = 0.31 |

**Table S9 note:** No significant interactions were found between pyruvate, lactate, or the predictive biomarker index and the covariates maternal depression, sibling depression, paternal depression, or neuroticism score. This, together with the visualisation of the general additive model (GAM) plots (Figure S5), suggest that the contributory predictive value of pyruvate and lactate do not dependent on the value of these other covariates.
