# Supplementary material for: A multivariate blood metabolite algorithm stably predicts risk and resilience to major depressive disorder in the general population
Source: eBioMedicine. 2023 Jun 14;93:104643. doi: 10.1016/j.ebiom.2023.104643 (PMC10275706; doi:10.1016/j.ebiom.2023.104643)

**Table S10: Summary of effect sizes for key biomarkers used to predict MDD susceptibility/resilience**


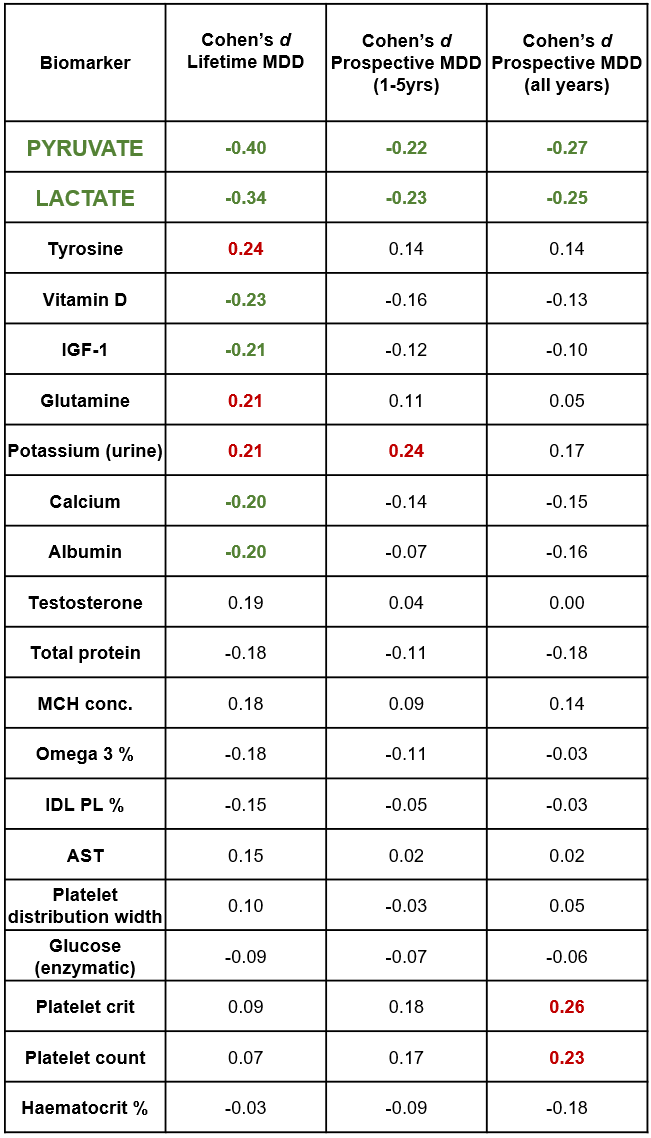

Supplement: Supplementary Table S10 [file mmc10.docx]
