# Supplementary material for: A multivariate blood metabolite algorithm stably predicts risk and resilience to major depressive disorder in the general population
Source: eBioMedicine. 2023 Jun 14;93:104643. doi: 10.1016/j.ebiom.2023.104643 (PMC10275706; doi:10.1016/j.ebiom.2023.104643)
Supplement: Supplementary Table S11 [file mmc11.docx]

| **TwinsUK cohort** | **Plasma Pyruvate** | **Plasma Lactate** |
| --- | --- | --- |
| Sample batch 1, visit 1  Blood sampling average 6.7yrs prior to depression questionnaire | Resilient (n=147): 0.046  Depressed (n=56): 0.050  p = 0.29 | Resilient: 1.53  Depressed: 1.52  p > 0.99 |
| Sample batch 2, visit 1 or 2  Blood sampling average 7.8yrs prior to depression questionnaire | Resilient: 0.056  Depressed: 0.063  p = 0.21 | Resilient: 1.30  Depressed: 1.36  p = 0.64 |
| Sample batch 2, visit 3  Blood sampling average 0.6yrs prior to depression questionnaire | **Resilient (n=87): 0.084**  **Depressed (n=27): 0.071**  ***p = 0.042** | Resilient (n=87): 1.34  Depressed (n=28): 1.24  p = 0.33 |

**Table S11: Summary statistics of plasma pyruvate and lactate levels in resilient and susceptible twins**
