# Supplementary material for: A multivariate blood metabolite algorithm stably predicts risk and resilience to major depressive disorder in the general population
Source: eBioMedicine. 2023 Jun 14;93:104643. doi: 10.1016/j.ebiom.2023.104643 (PMC10275706; doi:10.1016/j.ebiom.2023.104643)
Supplement: Supplementary Table S12 [file mmc12.docx]

**Table S12: ICD10 code counts by cohort**


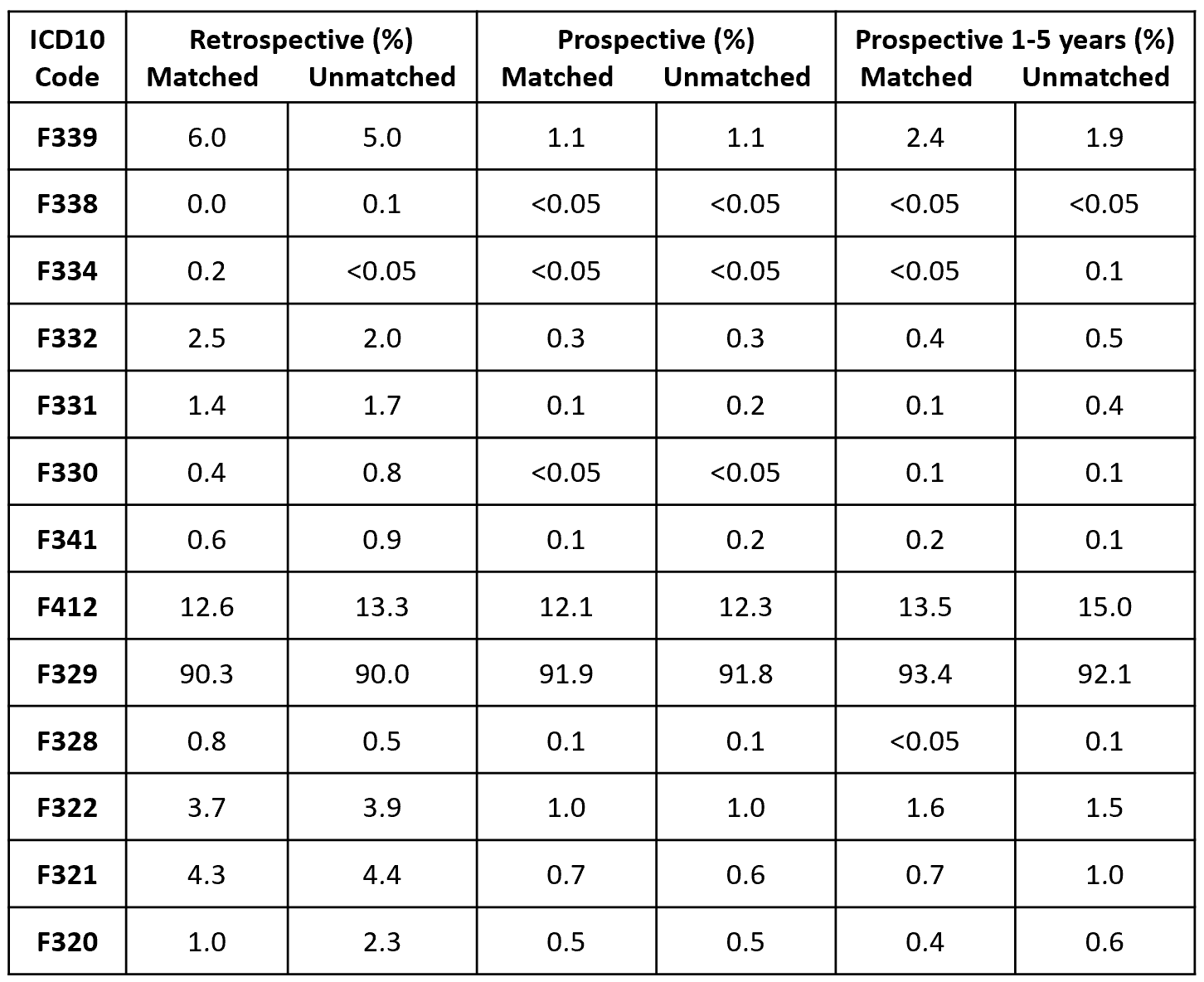


F339, recurrent depressive disorder, unspecified; F338, other recurrent depressive disorders; F334, recurrent depressive disorder, currently in remission; F332, recurrent depressive disorder, current episode severe without psychotic symptoms; F331, recurrent depressive disorder, current episode moderate; F330, recurrent depressive disorder, current episode mild; F341, dysthymia; F412, mixed anxiety and depressive disorder; F329, depressive episode, unspecified; F328, other depressive episodes; F322, severe depressive episode without psychotic symptoms; F321, moderate depressive episode; F320, mild depressive episode
