# Supplementary material for: A multivariate blood metabolite algorithm stably predicts risk and resilience to major depressive disorder in the general population
Source: eBioMedicine. 2023 Jun 14;93:104643. doi: 10.1016/j.ebiom.2023.104643 (PMC10275706; doi:10.1016/j.ebiom.2023.104643)
Supplement: Supplementary Table S14 [file mmc14.docx]

| **No. imputed covariates used for matching** | **Retrospective MDD  n = 491** | **Matched retrospective resilience  n = 491** | **Prospective MDD  n = 3,524** | **Matched prospective resilience  n = 3,524** |
| --- | --- | --- | --- | --- |
| **0** | 486 (99.0%) | 488 (99.4%) | 3510 (99.6%) | 3505 (99.5%) |
| **1** | 4 (0.81%) | 2 (0.41%) | 12 (0.34%) | 16 (0.45%) |
| **2** | 0 (0%) | 0 (0%) | 0 (0%) | 1 (<0.05%) |
| **3** | 0 (0%) | 1 (0.20%) | 0 (0%) | 2 (<0.05%) |
| **8** | 1 (0.20%) | 0 (0%) | 2 (0.06%) | 0 (0%) |

**Table S14: Summary of missing covariates that were imputed for the matched retrospective and matched prospective cohorts**
