# Supplementary material for: A multivariate blood metabolite algorithm stably predicts risk and resilience to major depressive disorder in the general population
Source: eBioMedicine. 2023 Jun 14;93:104643. doi: 10.1016/j.ebiom.2023.104643 (PMC10275706; doi:10.1016/j.ebiom.2023.104643)
Supplement: Supplementary Methods [file mmc15.docx]

**Supplementary Methods**

Resilient cohort derivation in UK Biobank

Informed by both the initial random forest and the wider literature, a subset of the control cohort was extracted and matched against both the retrospective and prospective MDD cohorts using an array of socioeconomic, demographic, and physical disease-associated features. The complete list, including detailed comparisons between cohorts, is shown in Supplementary Tables 4-5 (unmatched) and 6-8 (matched). Extrinsic factors such as social deprivation, employment status, and significant life stressors (financial, marital, trauma) were included, along with estimations of frailty (disease burden and self-reported health status), chronic pain, alcohol intake, smoking status, BMI, age, sex, and ethnicity. Baseline fluid intelligence was identical between resilient and susceptible individuals (Supplementary Table 13).

Due to the large number of covariates included in the matching formula, not all individuals with MDD were able to be matched. For the retrospective analysis, 491 MDD were matched against 491 controls, corresponding to 78% and 4% of each wider cohort, respectively.

For the 1–5-year prospective analysis, 1,083 MDD and 1,083 controls were matched, corresponding to 86% and 10% of each cohort, and for the entire prospective analysis, 3,524 MDD and 3,524 individuals were matched, corresponding to 74% and 32% of each wider cohort. The features used to predict the retrospective test set (Figure 3B) were used to predict each prospective cohort (1-5 years follow-up and all follow-up) from a model built using the entire retrospective cohort.

In the UK Biobank analysis, >99% of individuals in either the retrospective or prospective matched cohorts had no missing covariates used for matching (Supplementary Table 14). The subset of the TwinsUK cohort used for validation of the identified biomarkers did not include any missing data.

TwinsUK cohort derivation

To test the key findings obtained from the UK Biobank, we compared the levels of serum pyruvate and lactate in those who either did or did not have a lifetime history of depression in the TwinsUK biobank (field PH0001180), a predominately female cohort of middle age and older(15). Importantly, we selected for either resilience or susceptibility by only including individuals whose twin had the opposite answer to the same question, which would suggest either susceptibility (subject = depressed, twin = not depressed) or resilience (subject = not depressed, twin = depressed). 650 twins were found to be disparate for depressive symptoms, and we measured biomarkers in the blood of 115 individuals who had plasma metabolomic data at timepoint 3 which was most proximal to the date of the questionnaire (Supplementary Figure 7).

Supplementary statistical analyses

**Orthogonal partial least squares discriminant analysis**

To confirm the findings from the matched retrospective random forest analysis of MDD susceptibility and resilience using blood-based biomarkers, we analysed the same data using orthogonal partial least squares discriminant analysis (OPLS-DA) with feature selection. OPLS-DA was performed as previously described using the *ropls* package (1,2), with 10-fold cross validation and 10 x repetition with matched class sizes. This was performed in parallel on the Arcus-C supercomputing cluster using a hybrid MPI approach using OpenMPI 4.0.3 with OpenBLAS 0.3.9 and the DoParallel package (version 1.0.17) in R (4.0.2). During cross-validation, an extra feature selection step was incorporated whereby an logistic regression using the elastic net method was applied to the training set to reduce the number of features incorporated into the subsequent OPLS-DA method.

**General additive modelling and visualisation**

To aid in the visualisation of the independence of the effect of certain covariates, generalised additive models (GAM) were generated by (i) creating an outlier-rejected subset of the data of interest, where outliers were identified as being >1.5× the IQR, (ii) created via spline interpolated logistic regression in logit probability space (e.g. with the command gam(Class ~ s(Pyruvate) + Covariates, family=binomial) utilising the `mgcv` package in R) where the term Class denotes a subject’s classification as either belonging to the MDD or control groups; s() denotes a thin-plate spline created to smooth continuous variables, and then (iii) visualised via the vis.gam() function to provide a visual representation of the predictive power of each covariate in logit probability space on the z-axis, and covariate space on the x, y- axes. . These are representative contour plots of model predictions using GAMs, and were used to visually interpret the relationship between blood biomarkers and covariates such as a family history of severe depression and baseline neuroticism score.

**Post-processing quality control of NMR biomarker data**

Initial quality control of the nuclear magnetic resonance (NMR) biomarker data was performed according to the methods of Ritchie et al. (2021) to remove sources of technical variation including inter-spectrometer differences and drift over time within each spectrometer (3) via linear regression. This quality control was enabled through the use of internal controls, which were blinded duplicates of identical samples run throughout the course of the study. Methods are explained in detail in the paper by Ritchie et al. (3) and aim to eliminate the following sources of technical variation:

1. Duration of sample preparation
2. Position on plate (row and column position on 96 well plate independently contributed to sample variation)
3. Variation due to drift over time with a given spectrometer and between the 6 spectrometers used to analyse all samples
4. Removal of outliers considered to be contaminated with compounds of non-biological origin

Derived biomarkers (ratios and percentages) were recomputed following quality control.

By comparing glucose measurements from different aliquots and assays (enzymatic from aliquot 1, NMR from aliquot 3) we also show that higher glucose levels may have resulted in a higher rate of degradation (Figure S8). Nevertheless, serum glucose measured independently on the first aliquot was not significantly different between matched retrospective and prospective cohorts (Figure S8B,D). Furthermore, there were no significant differences in blood processing time for NMR analysis (Figure S9). There were also no significant group differences for the time spent fasting prior to blood sampling at recruitment, and the time of day when the blood was sampled at recruitment, between resilient and susceptible individuals (Figure S10).

**Supplementary References**

1. Radford-Smith DE, Selvaraj EA, Peters R, Orrell M, Bolon J, Anthony DC, et al. A novel serum metabolomic panel distinguishes IgG4-related sclerosing cholangitis from primary sclerosing cholangitis. Liver Int. 2022 Feb 7;

2. Thévenot EA, Roux A, Xu Y, Ezan E, Junot C. Analysis of the Human Adult Urinary Metabolome Variations with Age, Body Mass Index, and Gender by Implementing a Comprehensive Workflow for Univariate and OPLS Statistical Analyses. J Proteome Res. 2015 Aug 7;14(8):3322–35.

3. Ritchie SC, Surendran P, Karthikeyan S, Lambert SA, Bolton T, Pennells L, et al. Quality control and removal of technical variation of NMR metabolic biomarker data in ∼120,000 UK Biobank participants. medRxiv. 2021 Jan 1;2021.09.24.21264079.
